# A consensus-based classification workflow to determine genetically inferred ancestry from comprehensive genomic profiling of patients with solid tumors

**DOI:** 10.1093/bib/bbae557

**Published:** 2024-10-29

**Authors:** Zachary D Wallen, Mary K Nesline, Sarabjot Pabla, Shuang Gao, Erik Vanroey, Stephanie B Hastings, Heidi Ko, Kyle C Strickland, Rebecca A Previs, Shengle Zhang, Jeffrey M Conroy, Taylor J Jensen, Elizabeth George, Marcia Eisenberg, Brian Caveney, Pratheesh Sathyan, Shakti Ramkissoon, Eric A Severson

**Affiliations:** Medical Oncology, Labcorp Oncology, 6 Moore Dr., Durham, NC 27560, United States; Medical Oncology, Labcorp Oncology, 6 Moore Dr., Durham, NC 27560, United States; Medical Oncology, Labcorp Oncology, 6 Moore Dr., Durham, NC 27560, United States; Medical Oncology, Labcorp Oncology, 6 Moore Dr., Durham, NC 27560, United States; Medical Oncology, Labcorp Oncology, 6 Moore Dr., Durham, NC 27560, United States; Medical Oncology, Labcorp Oncology, 6 Moore Dr., Durham, NC 27560, United States; Medical Oncology, Labcorp Oncology, 6 Moore Dr., Durham, NC 27560, United States; Medical Oncology, Labcorp Oncology, 6 Moore Dr., Durham, NC 27560, United States; Department of Pathology, Duke University Medical Center, Duke Cancer Institute, 40 Duke Medicine Cir, Durham, NC 27710, United States; Medical Oncology, Labcorp Oncology, 6 Moore Dr., Durham, NC 27560, United States; Department of Obstetrics & Gynecology, Duke University Medical Center, Duke Cancer Institute, 40 Duke Medicine Cir, Durham, NC 27710, United States; Medical Oncology, Labcorp Oncology, 6 Moore Dr., Durham, NC 27560, United States; Medical Oncology, Labcorp Oncology, 6 Moore Dr., Durham, NC 27560, United States; Medical Oncology, Labcorp Oncology, 6 Moore Dr., Durham, NC 27560, United States; Labcorp, 531 South Spring Street, Burlington, NC 27215, United States; Labcorp, 531 South Spring Street, Burlington, NC 27215, United States; Labcorp, 531 South Spring Street, Burlington, NC 27215, United States; Oncology Medical Affairs, Illumina Inc, 5200 Illumina Way, San Diego, CA 92122, United States; Medical Oncology, Labcorp Oncology, 6 Moore Dr., Durham, NC 27560, United States; Department of Pathology, Wake Forest Comprehensive Cancer Center, Wake Forest School of Medicine, Medical Center Boulevard, Winston-Salem, NC 27109, United States; Medical Oncology, Labcorp Oncology, 6 Moore Dr., Durham, NC 27560, United States

**Keywords:** cancer, solid tumors, ancestry, genomic profiling, PCA, machine learning

## Abstract

Disparities in cancer diagnosis, treatment, and outcomes based on self-identified race and ethnicity (SIRE) are well documented, yet these variables have historically been excluded from clinical research. Without SIRE, genetic ancestry can be inferred using single-nucleotide polymorphisms (SNPs) detected from tumor DNA using comprehensive genomic profiling (CGP). However, factors inherent to CGP of tumor DNA increase the difficulty of identifying ancestry-informative SNPs, and current workflows for inferring genetic ancestry from CGP need improvements in key areas of the ancestry inference process. This study used genomic data from 4274 diverse reference subjects and CGP data from 491 patients with solid tumors and SIRE to develop and validate a workflow to obtain accurate genetically inferred ancestry (GIA) from CGP sequencing results. We use consensus-based classification to derive confident ancestral inferences from an expanded reference dataset covering eight world populations (African, Admixed American, Central Asian/Siberian, European, East Asian, Middle Eastern, Oceania, South Asian). Our GIA calls were highly concordant with SIRE (95%) and aligned well with reference populations of inferred ancestries. Further, our workflow could expand on SIRE by (i) detecting the ancestry of patients that usually lack appropriate racial categories, (ii) determining what patients have mixed ancestry, and (iii) resolving ancestries of patients in heterogeneous racial categories and who had missing SIRE. Accurate GIA provides needed information to enable ancestry-aware biomarker research, ensure the inclusion of underrepresented groups in clinical research, and increase the diverse representation of patient populations eligible for precision medicine therapies and trials.

## Introduction

Clinical research has historically relied on participants from a single self-identified race to mitigate the effects of underlying population differences. This has led to large gaps in our understanding of how cancer impacts diverse populations and reduces the generalizability of clinical research findings. Knowledge gaps and the limitations of research generalizability hinder cancer prevention efforts and optimization of patient treatment strategies for minority groups, leading to racial disparities in cancer outcomes [1–4]. Black and American Indian and Alaskan Native men have the highest overall cancer mortality rates, 18% higher than White men, and Black women have 40% higher breast cancer death rates than White women [5]. Patients from diverse communities face barriers at the individual, interpersonal, institutional, and policy levels that limit participation in clinical research [6]. Additionally, clinical studies seeking to understand patient differences based on genetic ancestry have relied on self-identified race and ethnicity (SIRE) as a proxy measure. However, SIRE is not consistently assessed using a universal standard and is often derived from questions with a small number of broad racial or ethnic categories that may or may not relate to a given patient, and concordance between SIRE and genetic ancestry can vary [7, 8]. These challenges, among others, have impeded advancements in including diverse populations in clinical research.

In place of SIRE, recent cancer studies have incorporated genetic ancestry information inferred from common single-nucleotide polymorphisms (SNPs) in a patient’s genome detected using different molecular approaches including whole-genome, whole-exome, whole-genome or targeted genotyping, targeted gene panels, and RNA sequencing [9]. Overall, genetic ancestry provides a more precise and objective assessment of an individual’s “biogeographical” ancestry compared to SIRE [7, 9–11]. However, accurate ancestry inference from tumor-derived DNA sequences is challenging due to the presence of somatic mutations, loss of heterozygosity, microsatellite instabilities, and other genomic abnormalities that can disrupt the accurate calling of germline SNPs [9]. Moreover, the targeted gene panels that typify comprehensive genomic profiling (CGP) assays in standard clinical use present additional challenges as they only measure a fraction of the genome, are enriched in genes prone to somatic mutations compared to other genomic regions, and target coding regions of genes where ancestry-informative variants are sparse. While sequencing or whole-genome genotyping of normal tissue can avoid some issues with tumor DNA [12], this testing is not yet feasible in the real-world cancer care setting, which is usually restricted to patient tumor biopsies taken during standard care.

Despite the challenges, clinical studies have attempted ancestry inference from CGP of patient tumors using a variety of workflows [9, 12–32]. These workflows typically involve (i) assignment of discrete ancestries through principal component analysis (PCA) of patient SNPs followed by classification with a machine learning algorithm and/or (ii) calculation of ancestral admixtures of patients providing quantitative measurements of ancestry. Both approaches involve training algorithms on genetic data from large, diverse reference populations, usually the 1000 Genomes (1000G) dataset [33]. There remain areas to be improved upon, however, two key areas being the inference method and reference dataset used for ancestry inference. Previous workflows have relied on separate use of one or two types of algorithms to make ancestry inferences, which opens their ancestry inferences to method choice bias, reduces the ability to detect “inconclusive” ancestry inferences, and potentially increases the likelihood of false positives. They also performed ancestry inferences using five (or less) of the main 1000G populations (African, European, Admixed American, East Asian, South Asian), which do not fully capture all the potential ancestries a patient might have.

Using genomic data from 4274 reference samples from eight world populations and 491 tumor samples from patients with SIRE data who underwent CGP testing, we developed and validated a new workflow to obtain accurate genetically inferred ancestry (GIA) from CGP sequencing results. Our workflow differs from previous workflows in several notable ways. In addition to the main 1000 Genome populations, our workflow includes inference of Middle Eastern, Central Asian/Siberian, and Oceania ancestry. This is a significant improvement upon previous workflows as the addition of these populations expands the geographical coverage of the reference dataset, allowing accurate ancestry inferences for individuals who would otherwise be incorrectly classified without a suitable reference population (e.g. individuals with Middle Eastern ancestry being classified as European or South Asian [34]). Furthermore, our workflow employs consensus-based classification that utilizes two principal component-based classification methods in conjunction with genetic admixture analysis to determine a consensus GIA call. This approach enhances the precision and reliability of ancestry assignments, reducing potential biases and uncertainties associated with relying solely on a single method. The workflow was designed to run on sequencing results from the TruSight® Oncology 500 (TSO 500) CGP assay within TSO 500 probe regions [35], but it can easily be extended to other CGP assays, allowing accurate GIA calls to be made across CGP assays.

Here, we provide details on the methods of our newly developed workflow, emphasizing points of improvement upon previous workflows, and report results from technically validating the workflow against SIRE data from real-world clinical patients. Additionally, we provide a brief review and comparison of previously published workflows to not only put characteristics of our workflow in the context of previous literature but also provide a resource for others, as a survey of published GIA workflows for CGP of tumor DNA has not been completed to date.

## Methods

### Ethical approval

Approval for this study was obtained from the Western Institutional Review Board Copernicus Group (WCG protocol # 1340120).

### Reference sample processing and data generation

To perform genetic ancestry inference, SNP data derived from a large, diverse reference dataset with known ancestry is needed. The most used reference dataset for ancestry inference is the 1000G project data, which currently includes deep (30×) whole-genome sequencing of lymphoblastoid cell lines from 3202 individuals covering five major geographical populations around the world [33], and is now part of the International Genome Sample Resource (IGSR; https://www.internationalgenome.org). Populations covered by 1000G include African (AFR), Admixed American (AMR; mainly Native Central and South American ancestry), East Asian (EAS), European (EUR), and South Asian (SAS) populations. Two additional diverse datasets included in the IGSR are the Human Genome Diversity Project (HGDP) [36] and the Simons Genome Diversity Project (SGDP) [37]. The HGDP and SGDP datasets together consist of 1072 deeply sequenced individuals (35×–43× on average) from the same five geographical populations as 1000G and, additionally, Middle Eastern (MEA), Central Asian/Siberian (CAS/SIB), and Oceania (OCN) populations [36, 37]. Including these datasets added 811 additional reference samples to the pre-existing 1000G populations and uniquely enabled inference of MEA, CAS/SIB, and OCN ancestry, which have yet to be included in previous workflows [9, 12–32]. A summary of the reference datasets and included populations used in our workflow can be found in Table 1 along with total reference sample numbers pre- and postprocessing. Individual-level data for reference samples can be found in Supplementary Table 1.

**Table 1:** Whole-genome sequenced reference samples used in the GIA workflow.

	**Datasets**				
**Populations**	**1000G**	**HGDP**	**SGDP**	**Total preprocessing**	**Total postprocessing**
African	893	88	35	1016	751
Admixed American	490	51	22	563	423
East Asian	585	170	51	806	728
European	633	137	43	813	705
South Asian	601	181	35	817	728
Middle Eastern	0	153	23	176	175
Central Asian/Siberian	0	23	27	50	50
Oceania	0	25	8	33	32
All populations	3202	828	244	4274	3592

1000G, 1000 Genomes; HGDP, Human Genome Diversity Project; SGDP, Simons Genome Diversity Project. The reduction of reference sample size from pre- to postprocessing was due to the exclusion of samples from related individuals.

**Figure 1:**
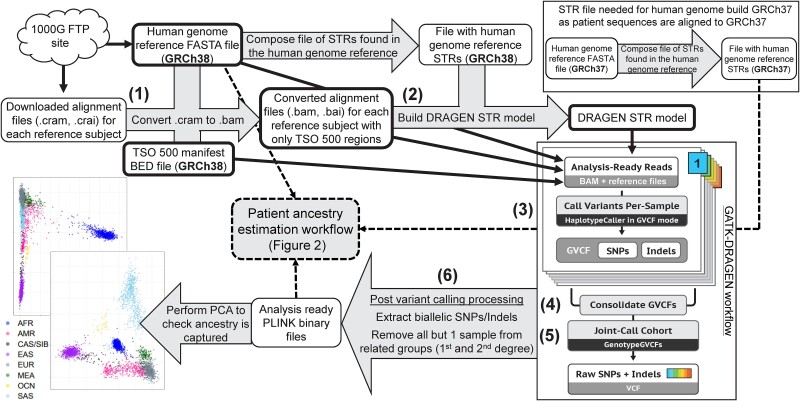
Reference sample processing and data generation. White boxes indicate files that have been previously generated or are produced as part of the workflow. Numbers correspond to the main steps of the reference sample processing: (1) CRAM to BAM file conversion, (2) DRAGEN STR model construction, (3) sample-level variant calling with GATK-DRAGEN, (4) sample-level variant call consolidation, (5) joint variant calling, and (6) postvariant call processing. Gray arrows or boxes represent processes that occur during the workflow. White boxes with bold borders correspond to inputs for variant calling. The figure for “GATK-DRAGEN workflow” was taken and modified from https://gatk.broadinstitute.org/hc/en-us/articles/360035535932-Germline-short-variant-discovery-SNPs-Indels. STR, short tandem repeat; TSO, TruSight® Oncology; SNP, single-nucleotide polymorphism; GVCF, genomic variant call format; PCA, principal component analysis; AFR, African; AMR, Admixed American; CAS/SIB, Central Asian/Siberian; EAS, East Asian; EUR, European; MEA, Middle Eastern; OCN, Oceania; SAS, South Asian.

A flowchart showing the general steps taken for reference sample processing and data generation is provided in Fig. 1. Compressed Reference-oriented Alignment Map (CRAM) files for the 1000G dataset and the HGDP and SGDP datasets were obtained from the 1000G Sequence Read Archive file transfer protocol (FTP) site (ftp://ftp.sra.ebi.ac.uk/vol1/run/) and 1000G data collections FTP site (ftp://ftp.1000genomes.ebi.ac.uk/vol1/ftp/data_collections/), respectively. Germline variant calling of SNPs and short insertion/deletions (Indels) utilizing GATK-DRAGEN was performed for reference samples using samtools v 1.7 [38] and GATK v 4.4.0.0 [39] targeting regions covered by the TSO 500 bait set. Germline variant calling involved the following steps: (i) CRAM to Binary Alignment Map (BAM) file conversion, (ii) DRAGEN short tandem repeat (STR) model construction (required when running GATK-DRAGEN), (iii) sample-level variant calling with GATK-DRAGEN, (iv) consolidation of single-sample variant calls per chromosome, (v) joint variant calling, and (vi) postvariant call processing. Details on how each step was performed are given below.

(1) CRAM to BAM file conversion. To convert CRAM files to BAM files for all datasets targeting only TSO 500 regions, the command `samtools view -b -T $GRCh38.fa -M -L $regions --write-index -o $file_name.bam $file_name.cram` was used where $file_name.cram was the input CRAM file of a reference sample, $regions was the TSO 500 manifest obtained directly from Illumina (San Diego, CA) that lists bait set regions in BED format, and $GRCh38.fa was the GRCh38 human genome reference used to create the CRAM files (ftp://ftp.1000genomes.ebi.ac.uk/vol1/ftp/technical/reference/GRCh38_reference_genome/GRCh38_full_analysis_set_plus_decoy_hla.fa).
(2) DRAGEN STR model construction. The STR model, required to run GATK-DRAGEN, was created using the command `gatk CalibrateDragstrModel --reference $GRCh38.fa --input $file_name.bam --intervals $regions --interval-padding 200 --str-table-path $GRCh38.STR.zip --output DRAGEN_STR_model.txt` where $GRCh38.STR.zip was a STR table file created with the GATK ComposeSTRTableFile function using the GRCh38 reference file as input.
(3) Sample-level variant calling with GATK-DRAGEN. SNP and Indel calling was performed using GATK’s HaplotypeCaller in DRAGEN mode by using the command `gatk HaplotypeCaller --reference $GRCh38.fa --input $file_name.bam --intervals $regions --interval-padding 200 ---output $file_name.g.vcf.gz --emit-ref-confidence GVCF --dragen-mode true --dragstr-params-path DRAGEN_STR_model.txt`, producing a genomic variant call format (GVCF) file with SNP and Indel calls for each sample.
(4) Sample-level variant call consolidation. Individual reference sample GVCFs were consolidated by chromosome with the command `gatk GenomicsDBImport --batch-size 50 --bypass-feature-reader true --consolidate true --intervals $chr --genomicsdb-workspace-path $chr.db --reference $GRCh38.fa --sample-name-map gvcf.sample_map`, producing a GenomicsDB datastore formatted file for each chromosome ($chr.db) that contains all SNPs and Indels called for that chromosome from each reference sample.
(5) Joint variant calling of reference samples was performed for each chromosome using the command `gatk GenotypeGVCFs --reference $GRCh38.fa --variant gendb://$chr.db --output $chr.vcf.gz`, producing one VCF per chromosome with final variant calls for all reference samples.
(6) Postvariant call processing. Per-chromosome VCFs were combined, sorted, and indexed using the `concat`, `sort`, and `index` functions from bcftools v 1.7-2 [38]. Using PLINK2 (December 2023 release) [40], VCFs were converted to PLINK binary file formats keeping only autosome and biallelic variants. Genetic relatedness (measured via KING-robust coefficients [41]) was calculated using PLINK2, with one reference sample from related groups retained (KING-robust coefficient > 0.09375, i.e. first- and second-degree relations). African populations with observed high within-population genetic similarity (Mbuti, Biaka, Julʼhoan/San, and Bantu in South Africa and Tswana; *N* = 52) [36, 37] were also removed before patient ancestry inference, as the inclusion of these populations skewed PCA results.

After variant calling and processing, SNP data for 3592 (84%) reference samples were available and incorporated into the GIA workflow.

### Genetically inferred ancestry workflow for inferring patient ancestry from comprehensive genomic profiling sequencing results

**Figure 2:**
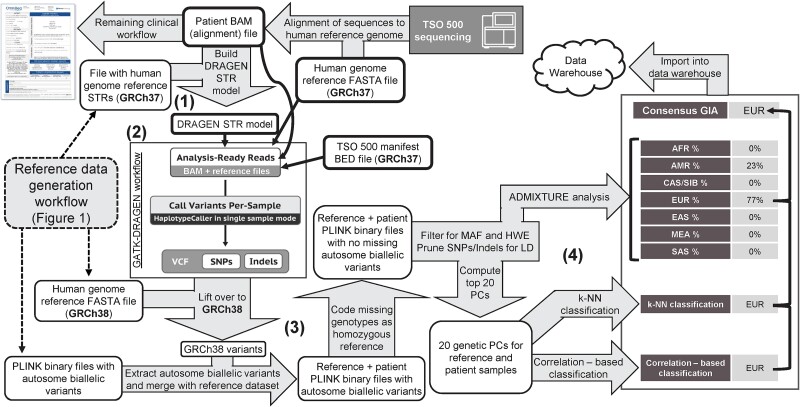
GIA workflow for inferring patient ancestry from TSO 500 sequencing results. White boxes indicate files that have been previously generated or are produced as part of the workflow. Numbers correspond to the main steps of the GIA workflow: (1) DRAGEN STR model construction, (2) sample-level variant calling with GATK-DRAGEN, (3) postvariant call processing and merging with reference dataset variants, and (4) GIA calling and consensus determination. Gray arrows and boxes represent processes that occur during the workflow. White boxes with bold borders correspond to inputs for variant calling. The figure for “GATK-DRAGEN workflow” was taken and modified from https://gatk.broadinstitute.org/hc/en-us/articles/360035535932-Germline-short-variant-discovery-SNPs-Indels. Of note, admixture analysis is only performed for seven (excluding the Oceania population) due to a bias noted during testing (see note in Methods). (B) STR, short tandem repeat; SNP, single-nucleotide polymorphism; VCF, variant call format; MAF, minor allele frequency; HWE, Hardy–Weinberg equilibrium; PC, principal component; k-NN, *k*-nearest neighbor; AFR, African; AMR, Admixed American; CAS/SIB, Central Asian/Siberian; EAS, East Asian; EUR, European; MEA, Middle Eastern; SAS, South Asian.

The GIA workflow described here (Fig. 2) was designed to take TSO 500 sequencing results within the validated OmniSeq® INSIGHT laboratory-developed test [35] as input, beginning with a sample-level DNA-sequence alignment file (GRCh37 build). From there, the GIA workflow includes four main steps: (i) DRAGEN STR model construction, (ii) sample-level variant calling with GATK-DRAGEN, (iii) postvariant call processing and merging with reference dataset variants, and (iv) performing ancestry inference from patient-reference merged data. Details on GIA workflow steps are provided below.

(1) DRAGEN STR model construction. The STR model is created using the command `gatk CalibrateDragstrModel --reference $GRCh37.fa --input $file_name.bam --intervals $regions --interval-padding 200 --str-table-path $GRCh37.STR.zip --output DRAGEN_STR_model.txt` where $file_name.bam is the BAM file with aligned tumor sequences from sequencing with TSO 500, $GRCh37.fa is the human reference genome FASTA file (as TSO 500 sequences are aligned with this genome build by default), $GRCh37.STR.zip is a STR table file created with the GATK ComposeSTRTableFile function using the GRCh37 reference file as input, and $regions are the TSO 500 bait regions in GRCh37 coordinates.
(2) Sample-level variant calling with GATK-DRAGEN. Commands for sample-level variant calling are the same as those used for calling variants of reference samples with the following modifications: GRCh37 reference genome and STR table files are used in place of GRCh38 files, patient sample-level variant calling is performed per chromosome to speed up processing, and no joint variant calling is performed as the workflow is performed for one patient sample at a time.
(3) Postvariant call processing and merging with reference dataset variants. Like processing of reference samples, per-chromosome VCFs are combined, sorted, and indexed using the `concat`, `sort`, and `index` functions from bcftools and then converted to PLINK binary file formats keeping only autosome and biallelic variants. However, before converting to PLINK binary files, to harmonize variant positions between patient variants (GRCh37 build) and reference variants (GRCh38 build), positions are updated to GRCh38 using the `LiftoverVcf` function from the Picard suite of tools (version 3.1.1; https://github.com/broadinstitute/picard). Patient sample variants are merged with reference sample variants using the `bmerge` function in PLINK v 1.9 (function not fully implemented in PLINK2 at the time of writing), automatically resolving any merging errors; then, missing genotypes are given the genotype of homozygous reference allele to avoid batch effects due to missingness. Lastly, using PLINK2, merged patient–reference variant data are filtered for variants with minor allele frequency (MAF) > 0.1% and Hardy–Weinberg equilibrium exact test *P*-value >1E-6 using the “midp” modifier to apply a mid-p adjustment to the exact test *P*-values [42]. This filtering step helps to ensure any detected pathogenic somatic mutations (which will only be seen in the patient, resulting in a very low MAF), and problematic SNPs are removed before ancestry inference.
(4) GIA calling and consensus determination. Three methods are used to derive GIA calls from the merged patient–reference variant data: two different principal component (PC)-based classification methods and admixture analysis. Then, a consensus GIA is derived from the outputs of all three methods.

For all three methods, variants are first pruned for linkage disequilibrium (LD) using PLINK2’s `indep-pairwise` function with a 100 variant sliding window and *r*^2^ threshold of 0.2.

For PC-based methods, LD-pruned variants are used to calculate the first 20 genetic PCs (via PLINK2) for patient and reference samples. The top 20 genetic PCs are used as input to two classification methods implemented in R (version 3.6.2): a standard *k*-nearest neighbor (k-NN) algorithm with *k* = 8 (via caret R package v 6.0.83) and a custom correlation-based algorithm. The k-NN algorithm is first trained on genetic PCs of reference samples; then, the trained model is used to infer an ancestry population for the patient. The correlation-based algorithm calculates the Pearson correlation between genetic PCs of the patient sample and PCs of every reference sample (3592 correlations), extracts the top 1% of calculated correlations, and predicts the patient’s ancestry population to be the reference population with the highest number of samples represented in the top correlations (see Supplementary Fig. 1 for example visualizations of outputs). The outputs from PC-based classifications are two discrete GIA calls (one of AFR, AMR, CAS/SIB, EAS, EUR, MEA, OCN, or SAS from k-NN and correlation-based classification). The correlation-based algorithm can also produce a “Mixed Ancestry” call if two or more populations are included in equal numbers among the top 1% of correlations; however, this outcome was never observed during technical validation.

For admixture analysis, LD-pruned variants are provided directly to the program ADMIXTURE v 1.3.0 [43, 44] along with a “population file” listing population designations of reference samples and a hyphen for the patient (symbolizing that the ancestry is unknown). Admixture analysis is performed using ADMIXTURE’s `--supervised` flag and setting *k* to equal the number of reference populations (seven populations; see note below). The output from ADMIXTURE includes predicted ancestry fractions of a patient being contributed to by each reference population. The population reaching an admixture fraction of >0.54 (majority of ancestry plus an additional 0.04 to account for noise) is considered the discrete GIA call when determining a consensus GIA call. Of note, data for Oceania reference samples (*N* = 32) are removed before running ADMIXTURE (hence *k* = 7) due to a bias noted during testing where ADMIXTURE would estimate every patient to have a fraction of Oceania ancestry, masking ancestry fractions from other populations. This bias might result from lower heterozygosity and higher LD previously shown in New Guinea–based Oceania populations [45] that make up much of the Oceania reference population used here. Oceania ancestry estimation is still made possible, however, through PC-based classification.

To derive a consensus call, two out of three methods are required to call the same GIA for a patient sample (Supplementary Fig. 2). If there is no alignment between the two PC-based classifications and all ancestry fractions are <0.54, the patient is given a consensus GIA call of “Mixed Ancestry.” If there is no alignment between any of the methods when a majority ancestry fraction is present for ADMIXTURE results, the patient is given a consensus GIA of “Inconclusive”; however, the individual method results are still included in the final output.

PC-based GIA calls, admixture fractions, and the consensus GIA call for a patient are the final outputs of the workflow.

### TruSight® Oncology sequencing of the technical validation cohort

DNA and RNA were co-extracted from formalin-fixed paraffin-embedded (FFPE) tissue specimens and submitted for library preparation and sequencing using the hybrid-capture-based TSO 500 assay (Illumina, San Diego, CA) as part of OmniSeq® INSIGHT (OmniSeq, Buffalo, NY). Within the TSO 500 assay, DNA sequencing with hybrid capture was used to detect small nucleotide variants in exonic regions of 523 genes (single- and multinucleotide substitutions, insertions, and deletions) and copy number variants in 59 genes (gains and losses), as well as analysis of microsatellite instability (MSI) and tumor mutational burden (TMB) genomic signatures. RNA sequencing with hybrid capture detects fusions and splice variants in 55 genes. Only DNA sequencing results were used as input to the GIA workflow for deriving GIA calls of the validation cohort.

### Technical validation of genetically inferred ancestry workflow

GIA was determined for a validation cohort of 504 patients who underwent CGP testing via TSO 500 at a reference laboratory (OmniSeq/Labcorp, Buffalo, NY, USA) during standard care (Table 2). Data for 484 patients (96%) were collected as part of the PREFER (PRospective rEgistry oF advanced stage cancER) clinico-genomic patient registry. Patients with advanced-stage solid cancers consented to participate in PREFER from multiple oncology practices focused on serving underrepresented populations [46]. The remaining 20 patients underwent CGP testing during standard care at an Alaskan-based facility that serves native Alaskan populations and were included to provide cases with American Indian or Alaska Native race, which were lacking in the PREFER registry. Of the 504 patients, 491 (97.4%) had available SIRE data to compare with GIA calls. These patients were assigned a reference population (one of AFR, AMR, CAS/SIB, EAS, EUR, and SAS) based on their SIRE data to provide a direct comparison to GIA calls (Table 2). Middle Eastern and Oceania populations were not assigned to patients as there were no appropriate racial or ethnic categories reported for these populations. The remaining 13 patients (2.6%) had unknown or missing SIRE data but were still included in ancestry inference as an example of how GIA can resolve missing SIRE data. Additionally, three patients had sequencing performed twice on the same tumor specimen (technical replicates), and six patients had sequencing performed on two different tumor specimens from two different tissues (biological replicates), which allowed us to assess the stability of GIA calls across sequencing runs on the same or different tumor specimens taken from different tissue locations. All calculations, analyses, and plotting for the technical validation of the GIA workflow were performed in R v 4.2.2 (https://www.r-project.org/).

**Table 2:** Validation cohort characteristics.

**Variable**	***N***	**Summary stats**
Total number of patients	504	
Self-reported race and ethnicity (*N*, %) [Given 1000 Genomes population]	491	
White [EUR]		367 (74.7%)
Black or African American [AFR]		82 (16.7%)
Hispanic or Latino [AMR]		19 (3.9%)
American Indian or Alaska Native [CAS/SIB]		20 (4.1%)
Asian—Indian [SAS]		2 (0.4%)
Asian—Vietnamese [EAS]		1 (0.2%)
Sex (*N*, %)	504	
Female		233 (46.2%)
Male		271 (53.8%)
Age, years (mean ± SD)	504	68.5 ± 12
Cancer type (*N*, %)	504	
Nonsmall cell lung cancer		138 (27.4%)
Colorectal cancer		79 (15.7%)
Breast cancer		57 (11.3%)
Pancreatic cancer		33 (6.5%)
Head and neck cancer		24 (4.8%)
Esophageal cancer		22 (4.4%)
Prostate cancer		22 (4.4%)
Neuroendocrine tumors		19 (3.8%)
Melanoma		16 (3.2%)
Stomach cancer		16 (3.2%)
Unknown primary cancer		15 (3%)
Liver and bile duct cancer		11 (2.2%)
Bladder cancer		8 (1.6%)
Kidney and renal pelvis cancer		7 (1.4%)
Sarcoma		7 (1.4%)
Uterine cancer		6 (1.2%)
Cervical cancer		5 (1%)
Ovarian cancer		5 (1%)
Small intestine cancer		5 (1%)
Other cancer		9 (1.8%)
Known clinical stage (*N*, %)	385	
Stage II		3 (0.8%)
Stage III		109 (28.3%)
Stage IV		273 (70.9%)
Tumor specimen location (*N*, %)	490	
Metastatic		164 (33.5%)
Primary		326 (66.5%)
TMB (mutations/Mb) (mean ± SD)	484	13.5 ± 28.4
TMB level (*N*, %)	484	
High (≥10)		138 (28.5%)
Not high (<10)		346 (71.5%)
MSI level (*N*, %)	489	
MSI high		13 (2.7%)
Stable		476 (97.3%)
Number of neoplastic cells per slide (*N*, %)	504	
<1000		120 (23.8%)
≥1000		112 (22.2%)
≥2000		272 (54%)
Tumor specimen cellularity (*N*, %)	504	
≤2		395 (78.4%)
>2		109 (21.6%)

*N*, number of patients with data; SD, standard deviation; AFR, African; AMR, Admixed American; CAS/SIB, Central Asian/Siberian; EAS, East Asian; EUR, European; SAS, South Asian; TMB, tumor mutational burden; MSI, microsatellite instability.

Several checks were performed independently of patients’ SIRE to ensure the robustness of GIA calls. Differences in the number of aligned reads between called GIA groups were assessed using the Wilcoxon rank-sum test (via the `wilcox.test` function in R) on log-transformed sequence read counts to ensure that GIA calls were not biased by the total number of sequence reads of a sample. The top two genetic PCs of patients were projected onto reference sample PCs to ensure that GIA calls of patients aligned with the reference group to which they were most genetically akin. To have one data point per reference sample for PC 1 and 2, the median was used as PCA was performed independently for each patient, resulting in many measures of PC 1 and 2 for each reference sample. Lastly, admixture fractions and their distributions within each GIA group were plotted to determine if called GIA groups had higher fractions of the appropriate populations.

GIA calls were compared to the SIRE of each patient to assess overall concordance (proportion of patients with matching GIA and SIRE) and classification performance metrics (sensitivity/recall, specificity, balanced accuracy, precision, F1-score). Patients with inferred ancestries without SIRE information were counted in the calculations as “misclassified” to assess the extent to which GIA deviates from SIRE due to the added resolution of appropriate ancestral populations that do not have a matching racial or ethnic category reported. Concordances between GIA calls and SIRE were also assessed for significant differences across patient tumor types, tissue characteristics (primary versus metastatic sites, number of neoplastic cells per specimen slide, tumor specimen cellularity), and genomic characteristics (TMB low/high, MSI status, presence or absence of copy number alterations or gene fusions/rearrangements) using Fisher’s exact test to ensure that these factors do not introduce any biases.

Classification performance metrics were calculated via the `confusionMatrix` function in the caret R package v 6.0.94 [47], specifying the mode to be “prec_recall.” Plotting of technical validation results was performed using ggplot2 v 3.4.0 (https://ggplot2.tidyverse.org/) and various packages to extend ggplot2 functionality (ggpubr v 0.5.0, ggalluvial v 0.12.5). For any statistical analyses, uncorrected *P*-values <.05 were considered statistically significant. All reported *P*-values were two-sided.

## Results and discussion

### Technical validation results of the genetically inferred ancestry workflow

#### Validation cohort characteristics

The validation cohort used for assessing the performance of the GIA workflow consisted of patient tumor samples from a spectrum of races and ethnicities, ages, cancer types, and genomic biomarker characteristics (Table 2). Most patients self-identified as White (74.7%) followed by Black or African American (16.7%), American Indian or Alaska Native (4.1%), Hispanic or Latino (3.9%), and Asian (0.6%). While “Asian” does not typically distinguish between East and South Asian individuals [48], patients who self-reported Asian race also included their ethnicities, so we could subclassify these patients as Asian—Indian (0.4%) or Asian—Vietnamese (0.2%). Of note, while there were 20 patients with American Indian or Alaska Native for their race, 52 patients self-identifying as White (14.1% of White patients) reported their ethnicity as “Native American” (Supplementary Table 2A). White (without Hispanic or Latino ethnicity), Black or African American, and American Indian or Alaska Native patients were given a reference population label of EUR, AFR, and CAS/SIB, respectively. Hispanic or Latino patients were given a reference population label of AMR. Typically, American Indian or Alaska Native–identifying individuals would be labeled as AMR along with Hispanic or Latino; however, the addition of the CAS/SIB reference group in our workflow allows us to distinguish between North and Central/South American–based Native American ancestry [49, 50]. Asian patients reporting an ethnicity of Indian or Vietnamese were given a reference population label of SAS or EAS, respectively. The validation patient cohort was relatively balanced for males and females. Most patients were older than 60 years with a mean age of 68.5 ± 12. Patients with nonsmall lung cancer accounted for almost one-third of the dataset (27.4%), followed by colorectal (15.7%), breast (11.3%), pancreatic (6.5%), head and neck (4.8%), and 15 other cancer types comprising <5% of the cohort each. Most patient tumor tissue samples were collected from primary sites (66.5%), had low TMB (<10 mutations/Mb, 71.5%), and were microsatellite stable (97.3%).

#### Genetically inferred ancestry workflow results for the validation cohort

**Figure 3:**
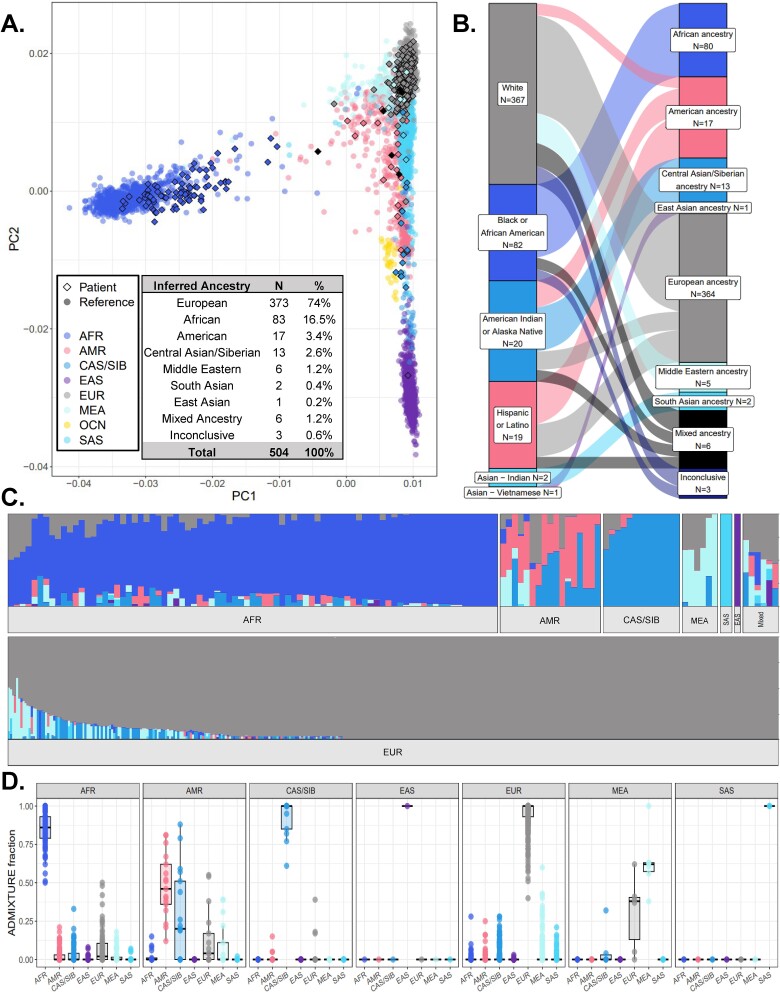
Results from performing GIA workflow for 504 patients whose tumors were tested via CGP as part of their standard care. (A) Projection of patient data points who had noninconclusive consensus GIA (*N* = 501) onto reference samples (*N* = 3592) within a combined patient PCA plot. Each point represents a unique reference sample (circles) or patient (diamonds). Points are colored by reference sample ancestral populations or if patients were determined to be of mixed ancestry (dark diamond points). Distances between points represent how similar genetic PCs were between a reference sample or patient compared to others in the plot. A full breakdown of GIA calls for patients including inconclusive patients is provided as a table within the PCA plot. (B) Relationship between patients’ SIRE and their consensus GIA call shown via Sankey chart (*N* = 491 patients with SIRE data). The size of nodes on each side of the chart and links in the middle represent the number of patients belonging to a group (nodes) or the number of patients of a certain race or ethnicity being classified into an ancestral group (links). For plot clarity, patient numbers were log-transformed before plotting. (C) Ancestral fractions for patients with noninconclusive consensus GIA, calculated via ADMIXTURE. Each bar represents what fraction of ancestry is contributed by each ancestral population for a unique patient, adding up to 1 (100%). (D) Distributions of ancestral fractions from ADMIXTURE within each GIA group. The bottom, middle, and top horizontal boundaries of each box in the box plots represent the first, second (median), and third quartiles of the data for a particular GIA group. The lines extending from the two ends of each box represent 1.5× outside the interquartile range. Points beyond the lines are considered outliers. AFR, African; AMR, Admixed American; CAS/SIB, Central Asian/Siberian; EAS, East Asian; EUR, European; MEA, Middle Eastern; OCN, Oceania; SAS, South Asian.

The GIA workflow was used to successfully obtain a consensus GIA for 501 patient samples (99.4%) from the validation cohort, while three patient samples were inconclusive for their consensus GIA (Fig. 3A, Supplementary Table 2A). All three technical replicates and six biological replicates had 100% concordance in GIA calls with only slight fluctuations in their ancestral fractions estimated via ADMIXTURE (Supplementary Table 2B). Tissue locations from which biological replicates were taken ranged from different areas of the same organ site (e.g. varying regions of the lung) to separate areas of the body (e.g. breast and small intestine) (Supplementary Table 2B), suggesting that reproducibility of GIA calls was not influenced by differences in tumor specimen tissue location.

Overall, the average number of aligned sequence reads per patient BAM file was 117 million, and no outlying GIA groups were observed in the number of aligned reads (Supplementary Fig. 3) showing that GIA calls overall were not biased by the total number of sequence reads of a sample. Failure to call a consensus GIA for three patient samples resulted from isolated instances of poor sequencing output, and therefore poor SNP calling, as sequence alignment files for patients with inconclusive GIA calls contained <12 000 sequences (8578 to 11 779 sequences) while the next-highest number of sequences was 11 million. Interestingly, all patient samples that resulted in an inconclusive consensus GIA had the same profiles among the three classification methods regardless of their SIRE: AMR for correlation-based classification, SAS for k-NN classification, and 100% CAS/SIB for admixture analysis (Supplementary Table 2A), which suggests that inconclusive results, at least those derived from low sequencing and SNP output, can be consistently caught.

When projected onto reference sample genetic PCs (Fig. 3A), patient data points overlapped with reference samples from their inferred ancestral population, which suggests that GIA calls overall were made successfully with no “off-target” inferences. Patients’ consensus GIA calls aligned as expected with their SIRE and expanded upon SIRE by (i) differentiating between those of European and Middle Eastern ancestry who reported as “White” and (ii) detecting the presence of mixed ancestry (Fig. 3B). GIA calls were also able to resolve heterogeneous racial categories with high ancestral admixture (e.g. American Indian or Alaska Native and Hispanic or Latino categories) (Fig. 3B). Ancestral fractions calculated via ADMIXTURE mirrored the consensus GIA calls (Fig. 3C) with the highest ancestral fractions within each consensus GIA group corresponding to the inferred ancestral population (Fig. 3D). It was interesting to note that while the highest median ancestral fraction of the AMR GIA group was AMR ancestry, this group also had high fractions of CAS/SIB ancestry (Fig. 3D), which may reflect expected Central Asian/Siberian ancestry in Central/South American–based Native American populations [49, 50]. All patients who self-identified as White and reported Native American as their ethnicity resulted in consensus GIA calls of EUR and had overall lower ancestry fractions from the AMR (0.4% ± 1.3%) and CAS/SIB (2.9% ± 14.1%) populations compared to patients identifying as American Indian or Alaska Native (AMR = 9.1% ± 15.8%, CAS/SIB = 75.9% ± 30.9%) or Hispanic or Latino (AMR = 37.9% ± 27.6%, CAS/SIB = 10.2% ± 17.8%) (Supplementary Table 2A). However, compared to White patients with no reported ethnicity, these patients had slightly, but significantly, higher AMR ancestry fractions (0.4% versus 0.3%, *P* = .01; Supplementary Fig. 4), suggesting that these patients potentially have Native American ancestry with higher proportions of European admixture.

#### Genetically inferred ancestry call performance

**Figure 4:**
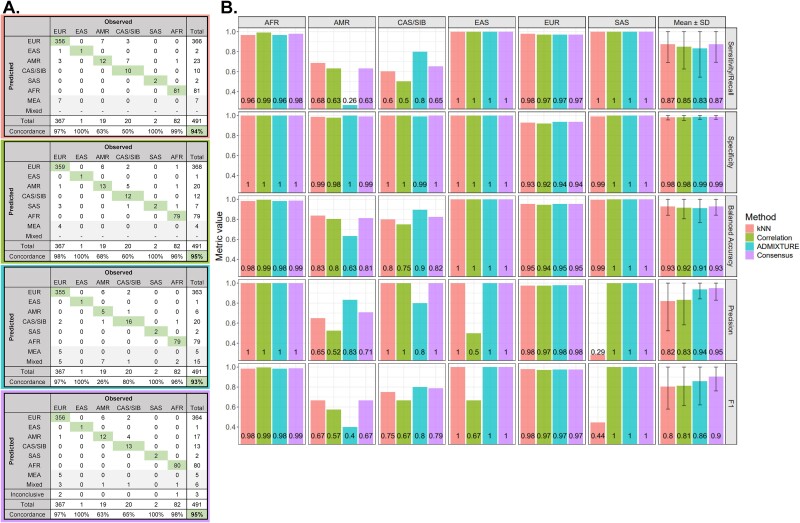
Classification performance of GIA workflow compared to patients’ SIRE. Classification performance for GIA classification methods was measured to assess how well GIA calls recapitulated the SIRE of patients. (A) Confusion matrices showing the relationship between SIRE (observed) and GIA (predicted) of each patient for k-NN (top row), PC correlation (second row), ADMIXTURE (third row), and consensus GIA (fourth row). Observed columns correspond to given 1000G ancestry populations based on patients’ self-identified race. Predicted rows correspond to GIA calls derived from the classification algorithms. Boxes in the diagonal of the confusion matrices indicate concordance between observed and predicted classifications aligned. Concordance percentages were calculated by summing the values in diagonal boxes and dividing by the total number of patients with SIRE data for each ancestry group and the total cohort (bold text). Mixed ancestry predictions did not apply to k-NN and PC correlation methods; therefore, those rows do not have values. MEA and Mixed classifications were only available with GIA calls. (B) Classification performance metrics are calculated from confusion matrices for individual classification methods and consensus GIA. Performance metrics were calculated for each ancestral population independently and then averaged across populations to get overall performance metrics (column “mean ± SD”). For all metrics, the higher the value, the better the method performed based on that metric. Performance metrics were not calculated for “Middle Eastern” or “Mixed Ancestry” groups as no self-identified race corresponding to those groups was recorded for patients. AFR, African; AMR, Admixed American; CAS/SIB, Central Asian/Siberian; EAS, East Asian; EUR, European; MEA, Middle Eastern; SAS, South Asian; SD, standard deviation.

GIA calls showed high concordance with SIRE (93%–95% for individual methods and 95% for consensus GIA calls; Fig. 4A) and resulted in a high classification performance when using SIRE as the ground truth (Fig. 4B). Classification metrics across all reference populations ranged from 0.8 to 1 depending on the classification method, with consensus GIA calls ranging from 0.87 (sensitivity/recall) to 0.99 (specificity). The classification performance of consensus GIA was equivalent to, or better than, the independent methods in the study, with the largest improvement seen for the F1 score (≤0.86 for independent methods versus 0.9 for consensus). The lowest concordance between consensus GIA calls and SIRE was seen for patients reporting as Hispanic or Latino (63%) and American Indian or Alaska Native (65%). A large portion of these patients (20.5%) resulted in GIA calls of EUR, which is an anticipated outcome due to the admixture of Native American and European populations [51]. However, consensus GIA calls had high specificity for these groups (0.99–1) and high precision for the CAS/SIB group, showing that the workflow errs on the side of less “false-positive” AMR and CAS/SIB ancestry calls. Precision was reduced for the AMR group (0.71) due to some American Indian or Alaska Native patients resulting in GIA calls of AMR, another expected outcome due to inherent relatedness between North and Central/South American–based Native Americans [52]. This also provides an example of how GIA is potentially capturing biological ancestry (i.e. Hispanic or Latino patients with Native American versus European lineages) compared to SIRE, which captures social and cultural constructs (i.e. labeled Hispanic or Latino regardless of lineage but based on where they currently reside, where they or their family immigrated from, etc.).

**Figure 5:**
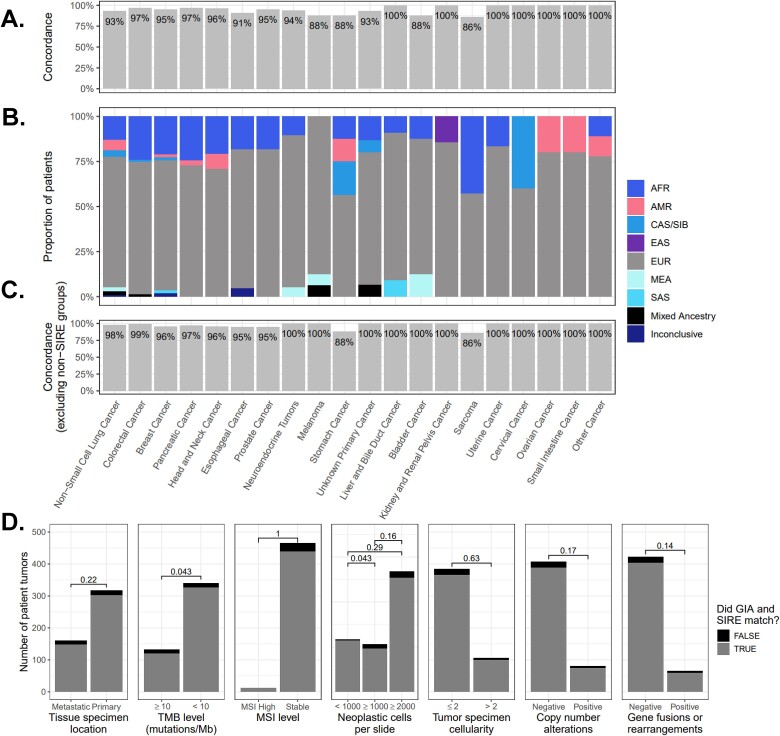
Concordance of consensus GIA classifications compared to a patient’s SIRE by tumor type and tumor characteristics. (A) Concordances of consensus GIA compared to SIRE for each tumor type. For each tumor type, concordance percentages were calculated by summing the number of times GIA matched with a patient’s SIRE and dividing by the total number of patients with SIRE data for each tumor type. (B) Proportion of GIA calls within each tumor type. (C) Concordances of consensus GIA compared to SIRE for each tumor type after excluding ancestry groups that were not covered by SIRE (MEA, Mixed Ancestry, Inconclusive). (D) Differences in GIA and SIRE concordance between different tumor tissue sites (primary versus metastatic tissue sites), levels of TMB and MSI, and amounts of tumor neoplastic cells and cellularity. Differences were tested using Fisher exact tests, and the values above bars represent the uncorrected *P*-values. AFR, African; AMR, Admixed American; CAS/SIB, Central Asian/Siberian; EAS, East Asian; EUR, European; MEA, Middle Eastern; SAS, South Asia.

Concordance between consensus GIA calls and SIRE varied by tumor type, with the lowest concordances seen among melanoma (81%), sarcomas (86%), stomach cancer (88%), and bladder cancer (88%) (Fig. 5A). Other tumor types ranged from 91% to 100% concordance between GIA calls and SIRE with an average concordance of 94.7% (Fig. 5A). Lower concordances for melanoma and bladder tumor types were mostly driven by higher proportions of GIA groups not covered by SIRE (i.e. MEA and Mixed Ancestry; Fig. 5B). After removing GIA groups not covered by SIRE, concordances for melanoma and bladder groups increased to 93% and 100%, respectively, and the overall average increased to 97.1% (Fig. 5C).

Concordance between consensus GIA calls and SIRE was not significantly influenced by whether a patient’s tumor was derived from a primary or metastatic site (*P* = .22), the number of neoplastic cells in the tumor specimen (*P* ≥ .1), tumor cellularity (*P* = .48), or genomic characteristics of the tumor including MSI status (*P* = 1), or presence or absence of copy number alterations (*P* = .29) or gene fusions/rearrangements (*P* = .08) (Fig. 5D). Cases that had low TMB (<10 mutations/Mb) had a slightly, but significantly, higher concordance between GIA calls and SIRE when compared to high TMB cases (96% versus 91%) (Fig. 5D). This suggests concordances are not biased by where a tumor specimen is derived from, if a tumor exhibits mismatch repair deficiency, or contains larger structural alterations or gene rearrangements; however, the number of somatic mutations in the tumor DNA may slightly influence concordances with SIRE.

### Comparison with previously published genetically inferred ancestry workflows

Here, we provide a brief review of previously published GIA workflows and compare their features to our GIA workflow. This not only helps to put characteristics of our workflow in the context of previously published workflows but also provides a resource for others, as a survey of published GIA workflows for CGP of tumor DNA is currently unavailable. Characteristics of our workflow and previously published workflows can be found in Table 3.

**Table 3:** Characteristics of the current and previous workflows for estimating genetic ancestry from CGP sequencing results.

**Study**	**PubMed ID**	**Year**	**CGP assay used in study**	**N genes in assay**	**Variant caller used**	**N SNPs used**	**N PCs used**	**Algorithm(s) used**	**Reference dataset(s) used**	**Concordance with SIRE**	**Ancestry populations inferred**								
											AFR	AMR	EAS	SAS	EUR	MEA	CAS/SIB	OCN	Mixed/Other
**Current workflow**																			
Wallen	–	2023	OmniSeq® INSIGHT (TruSight® Oncology 500)	523	DRAGEN - GATK	17,643 ± 2.9	20	Consensus of k-NN, PC correlation, ADMIXTURE	1000G, HGDP, SGDP	95%	**X**	**X**	**X**	**X**	**X**	**X**	**X**	**X**	**X**
**Previous workflows**																			
Carrot-Zhang ^A^	32396860	2020	FoundationOne® CDx	324	Proprietary algorithm	~40 000	5	Random forest, ADMIXTURE	1000G	87%–98%	**X**	**X**	**X**	**X**	**X**				**X**
Huang ^A^	33637877	2021	FoundationOne® CDx	324	Proprietary algorithm	~40 000	5	Random forest, ADMIXTURE	1000G	NR	**X**	**X**	**X**	**X**	**X**				**X**
Israel ^A^	34080753	2021	FoundationOne® CDx	324	Proprietary algorithm	~40 000	5	Random forest, ADMIXTURE	1000G	NR	**X**	**X**	**X**	**X**	**X**				**X**
Westphalen ^A^	34285332	2021	FoundationOne® CDx	324	Proprietary algorithm	~40 000	5	Random forest, ADMIXTURE	1000G	NR	**X**	**X**	**X**	**X**	**X**				**X**
Murugesan ^A^	34476330	2021	FoundationOne® CDx	324	Proprietary algorithm	~40 000	5	Random forest, ADMIXTURE	1000G	NR	**X**	**X**	**X**	**X**	**X**				**X**
Gusev ^B^	34749793	2021	Oncopanel (275, 300, 447)	275–447	STITCH (imputation algorithm)	1 million w/ impute	–	SNPWEIGHTS	1000G	~98%	**X**		**X**		**X**				
Brawley ^A^	34752134	2021	FoundationOne® CDx	324	Proprietary algorithm	~40 000	5	Random forest, ADMIXTURE	1000G	~93%*	**X**	**X**	**X**	**X**	**X**				**X**
Stopsack ^C^	34667026	2022	MSK-IMPACT® (341, 410, 468)	341–468	GATK Pileup	5072	–	ADMIXTURE	1000G	~52%–98%*	**X**	**X**	**X**	**X**	**X**				**X**
Lin ^A^	34998597	2022	FoundationOne® CDx	324	Proprietary algorithm	~40 000	5	Random forest, ADMIXTURE	1000G	NR	**X**	**X**	**X**	**X**	**X**				**X**
Myer ^A^	35176763	2022	FoundationOne® CDx	324	Proprietary algorithm	~40 000	10	Random forest, ADMIXTURE	1000G	NR	**X**	**X**	**X**	**X**	**X**				**X**
Adib ^B^	35428358	2022	Oncopanel (275, 300, 447), DUKE-F1-T5A, VICC-01-T5A, VICC-01-T7, MSK-IMPACT® (410 & 468)	275–468	STITCH (imputation algorithm)	1 million w/ impute	–	SNPWEIGHTS	1000G	90%	**X**		**X**		**X**				
Arora ^C^	36048199	2022	MSK-IMPACT® (HEME, 341, 410, 468, 505)	341–505	GATK Pileup	10 013	–	ADMIXTURE	1000G	High concordance, % not specified	**X**	**X**	**X**	**X**	**X**				**X**
Nassar ^B, C^	36179682	2022	Oncopanel (275, 300, 447), MSK-IMPACT® (341, 410, 468)	275–468	GATK, STITCH (imputation algorithm)	NR	4	Random forest, ADMIXTURE	1000G	90%	**X**	**X**	**X**	**X**	**X**				**X**
Chen ^A^	36446041	2022	FoundationOne® CDx	324	Proprietary algorithm	~40 000	10	Random forest, ADMIXTURE	1000G	NR	**X**	**X**	**X**	**X**	**X**				**X**
Peak ^A^	37196060	2023	FoundationOne® CDx	324	Proprietary algorithm	~40 000	NR	Random forest, ADMIXTURE	1000G	NR	**X**	**X**	**X**	**X**	**X**				**X**
Miyashita	37231433	2023	Tempus xT	648	Compare tumor DNA alignments to paired normal tissue alignments	654	–	ADMIXTURE	1000G	91%–97%	**X**	**X**	**X**	**X**	**X**				**X**
Sivakumar ^A^	37236698	2023	FoundationOne® CDx	324	Proprietary algorithm	~40 000	5	Random forest, ADMIXTURE	1000G	NR	**X**	**X**	**X**	**X**	**X**				**X**
Chehrazi-Raffle ^A^	37477913	2023	FoundationOne® CDx	324	Proprietary algorithm	~40 000	NR	Random forest, ADMIXTURE	1000G	NR	**X**	**X**	**X**	**X**	**X**				**X**
Kotecha ^C^	37864521	2023	MSK-IMPACT® (410, 468)	410–468	GATK Pileup	10 013	–	ADMIXTURE	1000G	80%–92%	**X**	**X**	**X**	**X**	**X**				**X**
Liu ^C^	37886874	2023	MSK-IMPACT® (410, 468)	410–468	GATK Pileup	10 013	–	ADMIXTURE	1000G	75%–86%	**X**	**X**	**X**	**X**	**X**				**X**

Superscript letters beside study names denote studies that used the same or similar workflows. A = Foundation Medicine workflow, B = Dana–Farber Cancer Institute and Harvard University workflow, C = MSK Cancer Center workflow. Cells with “X” represent ancestries that were attempted to be inferred for patients. Cells with “-” indicate characteristics that did not apply to the study. Numbers preceded by a “~” are approximated numbers due to reporting of numbers not being exact or having to be derived from inexact sources within the manuscript such as a table or figure NR = not reported in the study’s publication and could not be inferred based on referencing a previously published workflow. * = percentage derived from a figure. k-NN = *k*-nearest neighbor. AFR, African; AMR, Admixed American; CAS/SIB, Central Asian/Siberian; EAS, East Asian; EUR, European; OCN, Oceania; SAS, South Asian.

#### Study and workflow identification

Studies that utilized GIA workflows for CGP of tumor DNA were found using a PubMed search on 17 November 2023 with the following search criteria: (“cancer” AND “genetic ancestry” AND “sequencing”) OR (“genomic profiling” AND “ancestry”) OR (“sequencing panel” AND “ancestry”) OR (“sequencing assay” AND “ancestry”) and restricted to publications within the past 3 years (2020 to current). The PubMed search returned 66 abstracts, which were manually inspected to ensure that (i) the study utilized a validated, sequencing-based, targeted CGP assay to profile patient tumor DNA and (ii) performed a workflow for inferring genetic ancestry from the resulting CGP sequencing results. It was important to differentiate between those that designed a workflow using targeted CGP on tumor DNA and those that did not (e.g. use of normal patient DNA and/or whole-genome or -exome sequencing or genotyping) as the former would be more directly comparable to the currently described workflow and more applicable to real-world clinical testing. After excluding publications that did not meet these criteria, 20 remained.

Three GIA workflows were frequently used in studies from the same organizations with limited differences in workflow execution (study names with superscripts A, B, or C in Table 3). Organizations included Foundation Medicine Inc (Boston, MA, USA) [12–16, 19, 21, 22, 26, 27, 29, 30], Memorial Sloan Kettering (MSK) Cancer Center (New York, NY, USA) [20, 24, 31, 32], and Dana–Farber Cancer Institute/Harvard University (DFCI/HU) (Boston, MA, USA) [18, 23, 25]; therefore, these workflows will be referred to by their publishing institutions hereafter (Foundation, MSK, and DFCI/HU workflows). An additional workflow had only one study in the PubMed search results published jointly by the University of Chicago and Tempus Inc (UC/Tempus) (Chicago, IL, USA) [28].

#### Characteristics of previously published genetically inferred ancestry workflows

Previous published GIA workflows differed in terms of the CGP assay used, number of genes included in the assay, variant caller used, number of variants used to infer ancestry, whether genetic PCs were used in the inferences, the ancestry populations that were attempted to be inferred, and concordance of inferred ancestry with SIRE. Similarities between workflows were noted for the type of classification algorithms and reference data used to infer ancestry. The CGP assays used in the workflows included the FoundationOne® CDx (Foundation), Oncopanel (275, 300, 447 gene versions; DFCI/HU), MSK-IMPACT® (341, 410, 468, 505, HEME gene versions; MSK), the Tempus xT (UC/Tempus), and other custom panels. The number of genes targeted by CGP assays ranged from 275 to 648. The Foundation workflow utilized an undisclosed proprietary algorithm for calling SNPs from sequenced tumor DNA, while DFCI/HU, MSK, and UC/Tempus workflows used open-source software for calling SNPs, including a reference-free imputation algorithm called STITCH [53] (DFCI/HU workflow) and the widely used variant calling program GATK [39] (MSK workflow). The UC/Tempus workflow did not use a variant calling program but instead used the direct comparison of aligned tumor DNA with paired normal tissue to detect variants [28]. The number of nonimputed SNPs used for ancestry inference ranged from 654 (Tempus workflow) to ~40 000 (Foundation workflow), while studies using the DFCI/HU workflow reported upward of 1 million SNPs being called with imputation. Whether or not genetic PCs were used as input for ancestry inference was dependent on the type of algorithm used for assessing the genetic ancestry of patients. When workflows utilized classifier algorithms for predicting discrete ancestry populations (i.e. random forest; Foundation workflow), the top 5 or 10 genetic PCs were used as input for training the model and predicting patient ancestry. When workflows utilized machine learning algorithms for predicting ancestry fractions (i.e. ADMIXTURE or SNPWEIGHTS; MSK, DFCI/HU, UC/Tempus workflows), genetic PCs were not used, and variant data were inputted directly to the algorithms. All workflows utilized the 1000G dataset as a reference, and all, except for the DFCI/HU workflow, attempted to infer ancestry from the five main 1000G populations (AFR, AMR, EAS, EUR, SAS) and, additionally, mixed or other ancestry. The DFCI/HU workflow attempted inference of ancestries from African, Asian (mainly East Asian), and European populations only. Concordances between ancestry inferences and SIRE were reported in at least one study for each workflow and ranged from ~52% to 98% depending on methodology and the assessed ancestral population.

#### Comparison with previously published workflows

Our workflow (Table 3, top row) builds upon the previously published classification algorithms and reference datasets used to infer genetic ancestry. The CGP assay used in our workflow (TSO 500) targets a larger number of genes (523) than most CGP assays used previously, with only Tempus xT having more, allowing interrogation of more genomic regions. Our workflow not only utilizes an industry standard, open-source variant caller, GATK, as previously done by the MSK workflow, but also a state-of-the-art algorithm within the program (DRAGEN [54]) to call SNPs. The combination of using a broad CGP assay and robust variant calling algorithm allows our workflow to detect many SNPs (17 643 ± 2.9) within exonic regions of genes, resulting in one of the highest SNP numbers among previously published, nonimputation-based workflows (Foundation, MSK, UC/Tempus workflows).

Major differences and improvements between our workflow and previous workflows arise during the ancestry inference steps, from the generation of genetic PCs to the calling of the final ancestry population inference. One of the most obvious improvements of our workflow over previous workflows is the addition of more reference data, which increased the number of reference samples in the main 1000G populations and extended the number of populations available for ancestry inference (CAS/SIB, MEA, OCN). Extending the available reference populations requires additional resolution for inferring ancestry as more genetic correlation will inevitably exist between populations; therefore, our workflow doubled the number of genetic PCs used for inferring discrete ancestry populations compared to previous workflows (10 versus 20 PCs). The other major improvement upon previous workflows is the use of multiple algorithms to derive a consensus-based genetic ancestry inference. Until now, only one or two algorithms were used for estimating discrete ancestry populations (random forest) or ancestral fractions (ADMIXTURE, SNPWEIGHTS). If two algorithms were used, it consisted of one classification algorithm for estimating discrete ancestry populations and one algorithm for estimating ancestral fractions, and the results were analyzed separately (Foundation workflow). Using different, but complementary, algorithms to arrive at a consensus ancestry estimation helps to reduce method choice bias in ancestry inferences and allows for “inconclusive” calls when robust inferences cannot be made. Concordances between ancestry inferences and SIRE using our workflow (95%) were comparable to those previously reported by other workflows.

Of note, two previously published workflows reported higher numbers of SNPs being utilized in their workflows despite lower numbers of genes being included in the CGP assays: the Foundation (~40 000 SNPs) and DFCI/HU (1 million) workflows (Table 3, column “N SNPs used”). Two main areas that a workflow might deviate from ours to produce more SNPs include the algorithm being used to detect SNPs and the quality control of SNP genotypes. The Foundation workflow utilizes a proprietary variant calling algorithm and did not report any SNP quality control steps; therefore, we cannot comment on how this workflow deviates from our workflow concerning these areas. The DFCI/HU workflow utilizes an imputation-based algorithm (STITCH) for producing SNP genotype calls. An imputation algorithm takes a limited number of observed genotypes for SNPs detected via sequencing or genotyping and attempts to impute missing genotypes for detected and nondetected SNPs by comparing detected genotypes for an individual with a larger reference dataset or other individuals within the same dataset. This results in many more SNPs being included in the final dataset but has the caveat that the majority of SNPs will have imputed genotypes (i.e. an algorithmic estimation of what SNP genotype an individual would have if it were detected through sequencing or genotyping). This may produce artificially biased or incorrect downstream results depending on the type, size, and composition of the reference dataset used and the performance of the imputation algorithm. Our workflow consistently detected sufficient numbers of ancestry informative SNPs without the use of imputation; therefore, while our workflow utilized fewer SNPs than what is achieved through imputation, it still produced accurate calls without the threat of introducing artifacts that may arise from using imputed data.

## Limitations and future directions

While our GIA workflow provides several improvements over previously published workflows, certain limitations still exist in the workflow and its validation that will be improved upon with future work.

A consistent area of difficulty for ancestry estimation workflows is distinguishing individuals with Native American ancestry (AMR and CAS/SIB ancestry groups) from those with European ancestry (EUR ancestry group) as (i) a high degree of admixture is usually present between these two populations [51] and (ii) current publicly available reference datasets do not have adequate representation of North American-based Native American populations (Supplementary Table 1). Difficulty in resolving AMR and CAS/SIB versus EUR ancestry was observed with our workflow as well, with the AMR and CAS/SIB group having the lowest concordance between GIA and SIRE due to a large portion of Hispanic or Latino and American Indian or Alaska Native patients being estimated to have majority EUR ancestry. We found that our workflow errs on the side of fewer “false-positive” AMR and CAS/SIB ancestry calls at the expense of potentially missing some individuals with AMR and CAS/SIB ancestry, as specificity was high for both groups and precision was high for the CAS/SIB group with lower sensitivity/recall than other ancestry groups. This outcome is desired over having high sensitivity/recall but low specificity and precision as it will provide cleaner groups for statistical analyses and more robust AMR and CAS/SIB associations. Lower precision was observed for the AMR group compared to the CAS/SIB group as 20% of American Indian or Alaska Native patients resulted in AMR GIA calls, which is expected due to the close genetic relatedness of North and Central/South American–based Native Americans [52] and the AMR reference group being much larger than that of the CAS/SIB reference group (*N* = 423 versus 50, Table 1). Increasing the resolution for Native American ancestries, especially North American-based Native American ancestries, will require adding additional reference samples from these populations, which is a planned future direction for this workflow.

The validation dataset used to validate the workflow contains a diverse representation of patients from different racial and ethnic backgrounds but is not as large as some validation datasets reportedly used with other workflows [55]. Unfortunately, the size of the validation dataset is limited to the number currently reported in this manuscript as patient data were derived from a reference laboratory (OmniSeq/Labcorp) where extended patient demographics, including SIRE, are not routinely collected. We performed a literature search for published studies that included both TSO 500 sequencing and SIRE data to find external, independent datasets to include in the validation; however, no studies were found using the search criteria (“TSO500” OR “TSO 500” OR “TruSight Oncology”) AND “race” on PubMed or Google Scholar. This reflects a large gap in the literature concerning the landscape of TSO 500 testing of tumors from diverse populations, which our workflow will hopefully help to alleviate in future clinical studies.

Benchmarking against existing methods is an important step in introducing new methodologies or protocols. Unfortunately, we were unable to perform benchmarking of our GIA workflow against the previously published workflows summarized in the present study (Table 3) as they have not been made publicly available (Foundation, MSK, UC/Tempus workflows), use both paired normal and tumor sequences (Tempus workflow), or do not infer the full 1000G reference populations (DFCI/HU workflow). Instead, we had to rely on reported performance assessments by the groups authoring the GIA workflows. This reflects a deficiency in open-source tools for assessing GIA from CGP sequencing results, which we hope to absolve by making a docker image of our workflow publicly available (see Data Availability).

Finally, the current manuscript was designed as an introduction to and technical validation of our GIA workflow and was not powered for actual testing of associations between clinical and tumor characteristics of patients and different ancestries. A formal clinical study with a larger sample size is planned as a future direction for this workflow as a “clinical validation” study.

## Conclusion

Using genomic data from 4274 reference samples from eight geographical populations and 491 tumor samples from patients with SIRE data who underwent CGP testing, we developed and validated a new workflow to obtain accurate GIA. Our workflow improves upon previous workflows by expanding the pool of available reference populations, allowing for more comprehensive ancestry inferences, and utilizing consensus-based classification to obtain an accurate and robust GIA call. Our GIA workflow had high concordance with patients’ SIRE and could expand on SIRE by (i) detecting the ancestry of patients that usually lack appropriate racial categories, (ii) determining what patients have mixed ancestry, and (iii) resolving ancestries of patients in heterogeneous racial categories and who had missing SIRE. The workflow was designed to run on sequencing results from the TSO 500 CGP assay but can readily be extended to other CGP assays, allowing accurate GIA calls to be made across different tests from tumor DNA. Accurate GIA data provide needed information to enable ancestry-aware biomarker research, which can help mitigate cancer outcome disparities, ensure the inclusion of underrepresented groups in clinical research, and help findings be more representative of real-world patient populations with disease enabling targeted therapies and clinical trials to benefit all populations with disease.

**Key Points**

- Disparities in cancer diagnosis, treatment, and outcomes based on self-identified race and ethnicity are well documented, yet these variables have historically been excluded from clinical research.
- Genetic ancestry of a patient can be used as a proxy to self-identified race and ethnicity and can be inferred using comprehensive genomic profiling results from a patient’s tumor.
- We developed a workflow to accurately infer the genetic ancestry of patients from targeted sequencing of their tumor specimens.
- We improved upon previous workflows by increasing the number of inferred reference populations and utilizing consensus-based classification to obtain accurate and robust ancestry inferences.
- Accurate ancestry inference provides needed information to enable ancestry-aware biomarker research.

## Supplementary Material

Supplementary_Figures_bbae557

Supplementary_Table_1_bbae557

Supplementary_Table_2_bbae557

Supplementary_Code_bbae557

## Data Availability

The workflow, reference data, and required programs needed to perform the workflow have been incorporated into a docker container image that can be accessed on Docker Hub at the following repository: https://hub.docker.com/repository/docker/zwallen/tso500gia/general. See the repository page for details on how to run the workflow and required inputs. The 1000G, HGDP, and SGDP alignment data were accessed from publicly available FTP sites: ftp://ftp.sra.ebi.ac.uk/vol1/run/ (1000G) and ftp://ftp.1000genomes.ebi.ac.uk/vol1/ftp/data_collections/ (HGDP and SGDP). The human genome GRCh38 build reference FASTA used for aligning the 1000G, HGDP, and SGDP sequences are publicly available: ftp://ftp.1000genomes.ebi.ac.uk/vol1/ftp/technical/reference/GRCh38_reference_genome/GRCh38_full_analysis_set_plus_decoy_hla.fa. The GRCh37 build of the human genome reference used here is publicly available from Illumina (https://ilmn-dragen-giab-samples.s3.amazonaws.com/FASTA/GRCh37.fa). Individual-level population data for reference samples are provided in Supplementary Table 1. Individual-level race/ethnicity and GIA calls for the patient validation cohort are provided in Supplementary Table 2. R code and R session information for the calculations, analyses, and plotting used for the technical validation of the workflow can be found in the Supplement of the manuscript. Other data used in the validation analyses can be provided to the corresponding author upon reasonable request.
