# Supplementary material for: A consensus-based classification workflow to determine genetically inferred ancestry from comprehensive genomic profiling of patients with solid tumors
Source: Brief Bioinform. 2024 Oct 29;25(6):bbae557. doi: 10.1093/bib/bbae557 (PMC11521331; doi:10.1093/bib/bbae557)
Supplement: Supplementary_Figures_bbae557 [file supplementary_figures_bbae557.docx]

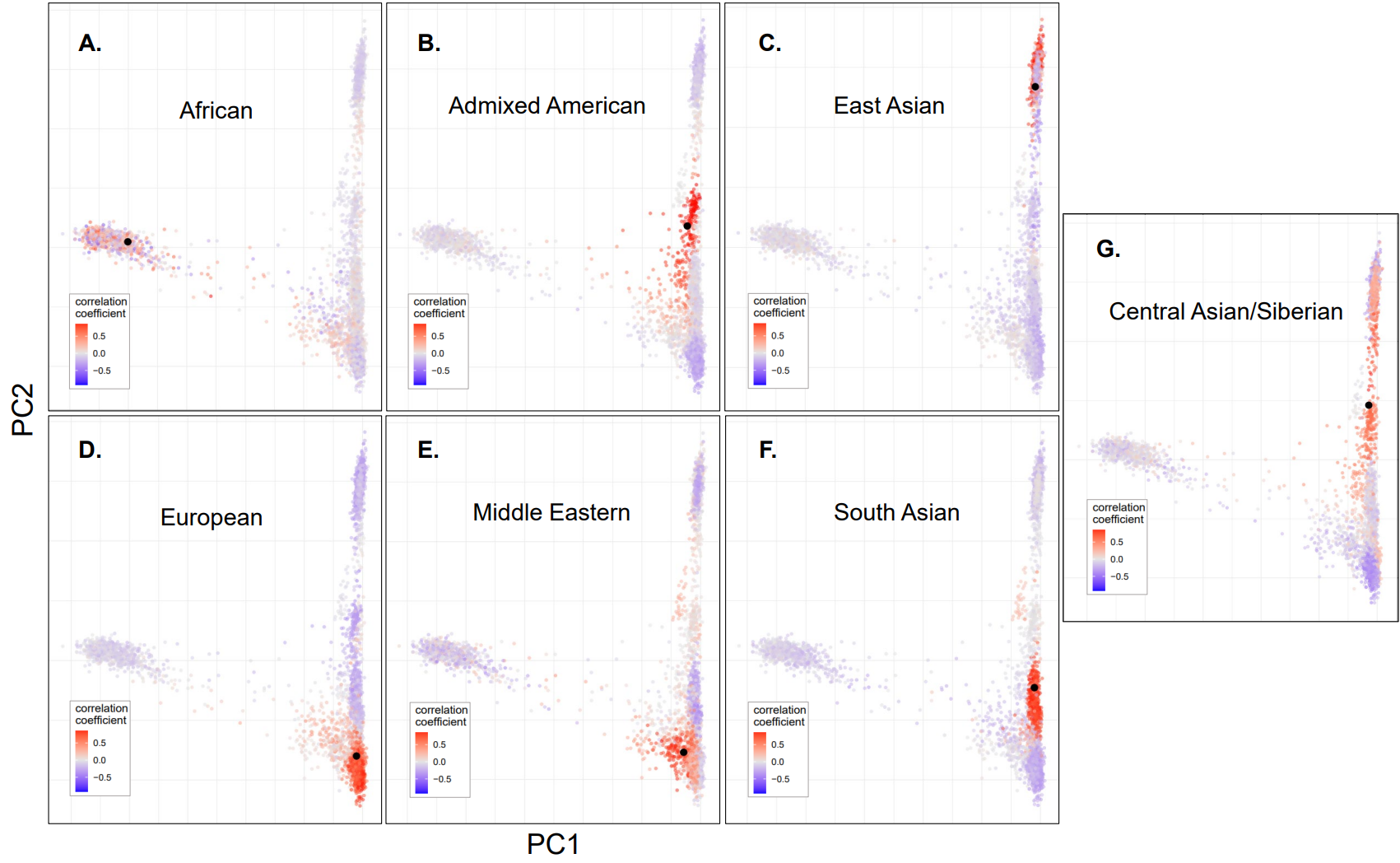


**Supplementary Figure 1.** Representative plots for each ancestry group that show examples of results derived from the principal component (PC) correlation-based algorithm. The top 20 genetic PCs of each patient derived from PCA with reference samples were correlated with the top 20 genetic PCs of each reference sample using Pearson correlation. Plots of PC1 and 2 were then annotated with the correlation coefficients to determine how well the correlation strengths captured the patient’s relationship with other reference samples in the PCA. Plots for the **(A)** African, **(B)** Admixed American, **(C)** East Asian, **(D)** European, **(E)** Middle Eastern, **(F)** South Asian, and **(G)** Central Asian/Siberian ancestry groups are shown here for representative patients of each group. Each point represents a unique reference sample (N = 3,592) or patient tumor sample. Points colored by correlation coefficient correspond to reference samples, while black points correspond to the representative patient. No consensus GIA calls relating to Oceania ancestry were made during technical validation of the GIA workflow, therefore, representative plots for these populations are not shown.


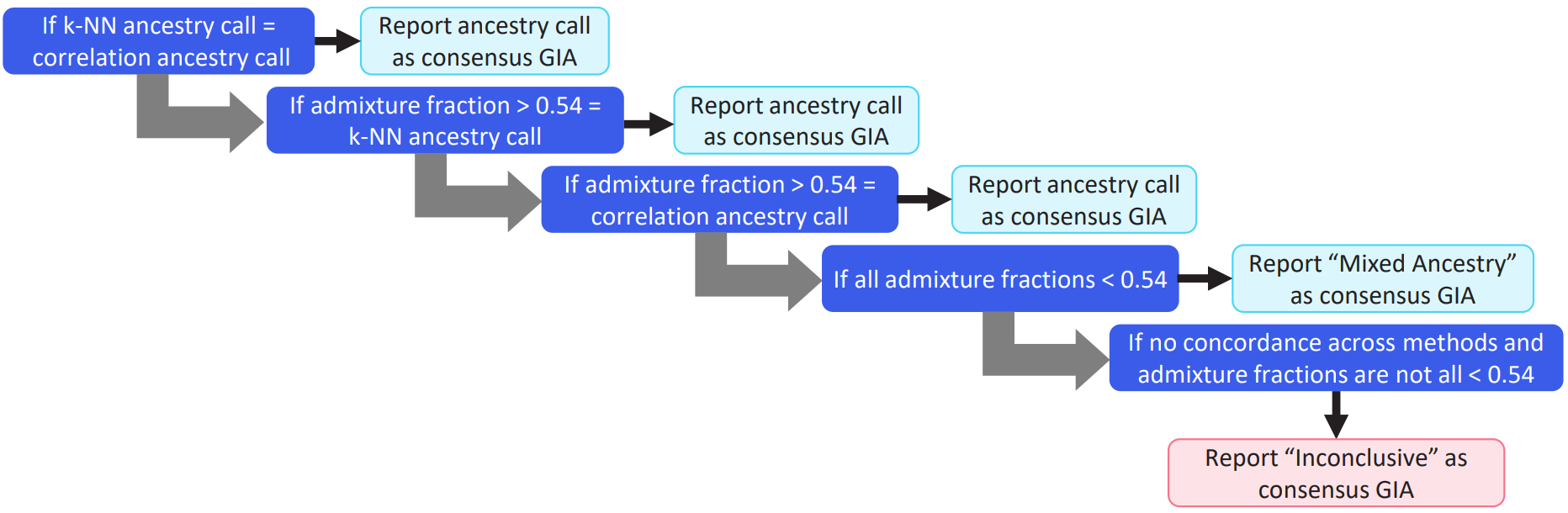


**Supplementary Figure 2.** The process for determining a consensus genetically inferred ancestry (GIA) for a patient based on GIA calls from the three individual classification methods (k-NN, PC correlation, admixture analysis). Dark blue boxes correspond to conditions in the consensus determination process. Light blue or pink boxes correspond to a consensus GIA output that results from a certain condition being met.


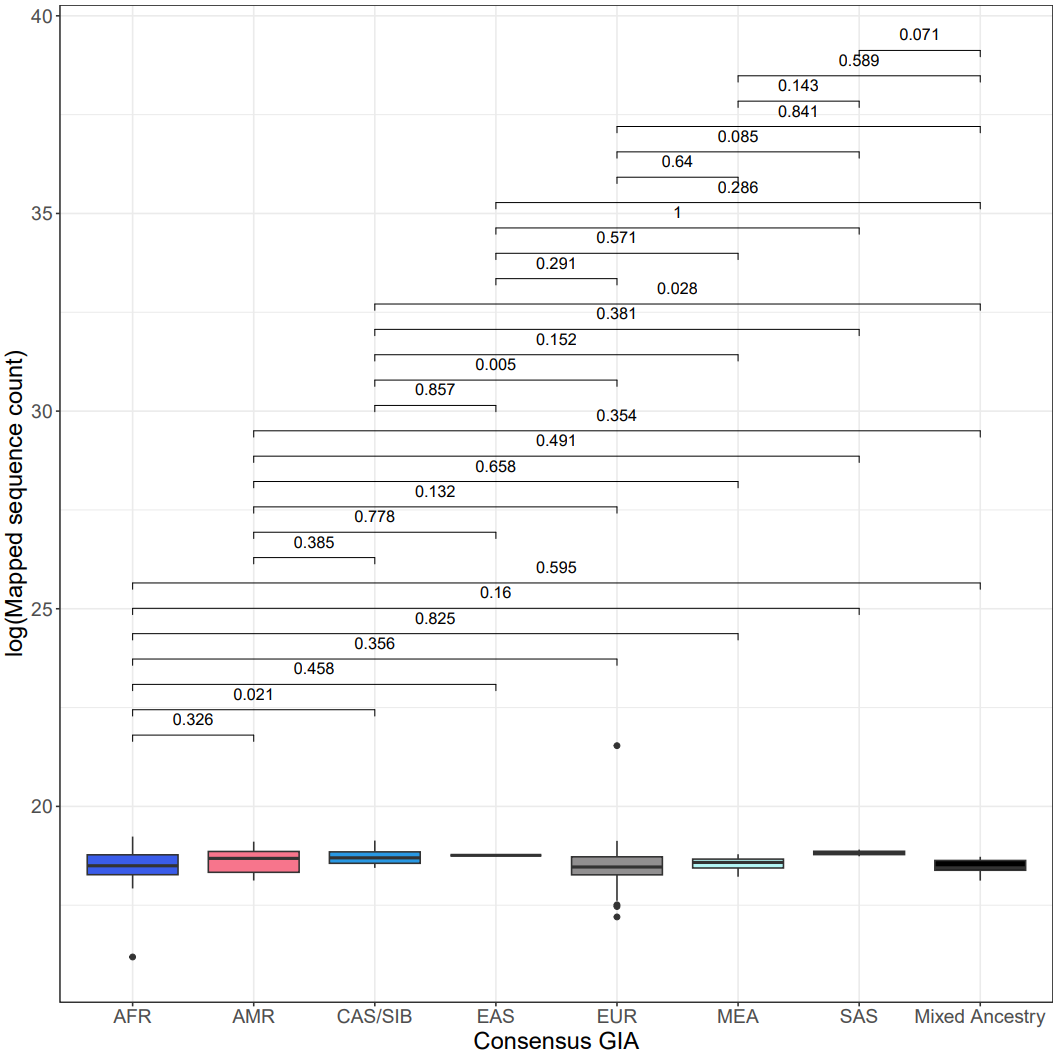


**Supplementary Figure 3.** Distributions of mapped sequences of patients within each genetically inferred ancestry (GIA) group. Differences in mapped sequences between consensus GIA groups were assessed using the Wilcoxon rank-sum test on log-transformed mapped sequence counts. Uncorrected P-values from testing are shown above each bar. The bottom, middle, and top horizontal boundaries of each box in the box plots represent the first, second (median), and third quartiles of the data for a particular GIA group. The lines extending from the two ends of each box represent 1.5x outside the interquartile range. Points beyond the lines are considered outliers. AFR, African; AMR, Admixed American; CAS/SIB, Central Asian/Siberian; EAS, East Asian; EUR, European; MEA, Middle Eastern; SAS, South Asian


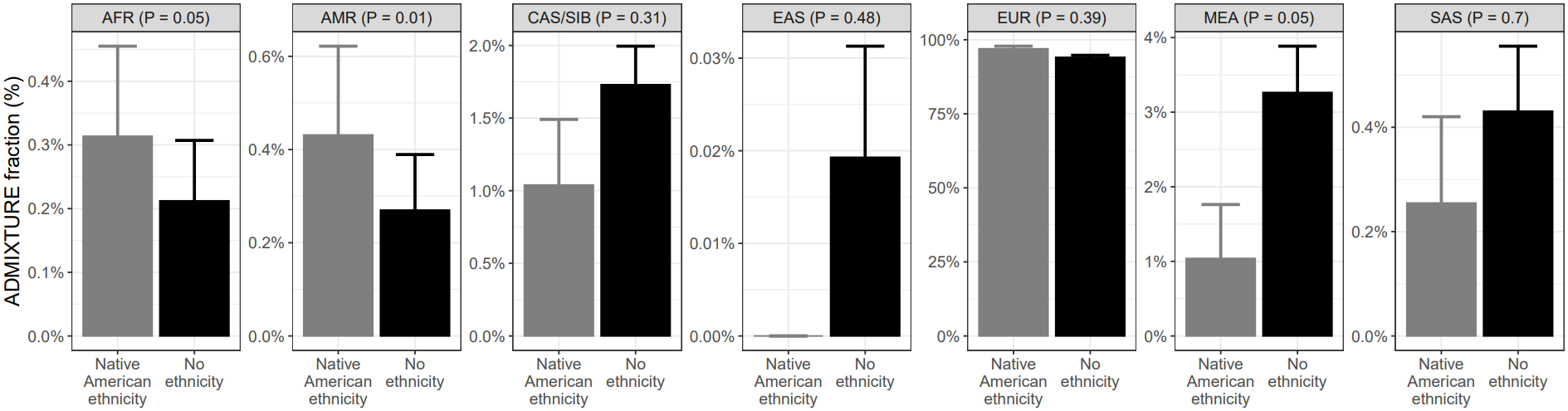


**Supplementary Figure 4.** Differences in overall ancestry fractions between White patients with no reported ethnicity and White patients reporting their ethnicity as Native American. Each column represents the mean of each group and error bars extending from columns represent the standard error of the mean. Differences between groups were tested using the Wilcoxon rank-sum test and resulting P-values are shown above each plot in parentheses. AFR, African; AMR, Admixed American; CAS/SIB, Central Asian/Siberian; EAS, East Asian; EUR, European; MEA, Middle Eastern; SAS, South Asian; P, P-value from Wilcoxon rank-sum test.
