# Supplementary material for: A consensus-based classification workflow to determine genetically inferred ancestry from comprehensive genomic profiling of patients with solid tumors
Source: Brief Bioinform. 2024 Oct 29;25(6):bbae557. doi: 10.1093/bib/bbae557 (PMC11521331; doi:10.1093/bib/bbae557)
Supplement: Supplementary_Code_bbae557 [file supplementary_code_bbae557.pdf]

TruSight Oncology 500 (TSO500) genetically inferred ancestry (GIA) workflow validation

Zachary D Wallen

October 15, 2024

Table of contents

1 Setting up the R environment **2**
1.1 Load required R packages . . . 2
1.2 Create excel styles used for formatting output . . . 2

2 Statistical analysis **2**
2.1 Import and prepare data . . . 2
2.2 Technical validation . . . 6
2.2.1 Patient characteristics of validation cohort . . . 6
2.2.2 Sequence coverage . . . 14
2.2.3 Technical validation results . . . 14
2.2.3.1 Relationship between self-reported race/ethnicity and consensus GIA calls . . . 14
2.2.3.2 Projection of patient genetic PCs on reference sample PCs . . . 18
2.2.3.3 ADMIXTURE ancestral fractions . . . 20
2.2.3.4 Performance metrics of GIA classification compared to self-reported race/ethnicity 23
2.2.3.5 Concordance of GIA classification compared to self-reported race/ethnicity by cancer type and tumor characteristics . . . 29

3 R session information **34**

1 Setting up the R environment

1.1 Load required R packages

```
library(plyr)
library(readxl)
library(tibble)
library(openxlsx)
library(foreach)
library(kableExtra)
library(ggplot2)
library(ggpubr)
library(grid)
library(ggalluvial)
library(caret)
```

1.2 Create excel styles used for formatting output

```
bold <- createStyle(textDecoration = "bold")
left_just <- createStyle(halign = "left", valign = "center", wrapText = TRUE)
right_just <- createStyle(halign = "right", valign = "center", wrapText = TRUE)
center <- createStyle(halign = "center", valign = "center", wrapText = TRUE)
horizontal_border_med <- createStyle(border = "top", borderStyle = "medium")
horizontal_border_thin <- createStyle(border = "top", borderStyle = "thin")
percentage <- createStyle(numFmt = "PERCENTAGE")
labcorp.col <- c(
    "#3a5ce9", "#4cd5f7", "#f7758c", "#918f90",
    "#1a2188", "#2998e3", "#6c2fac", "#b4f6f5"
)
```

2 Statistical analysis

2.1 Import and prepare data

```
# read data
ref.pops <- read.table(
    "1000G_HGDP_SGDP_subjects.txt",
    header = TRUE, sep = "\t", check.names = FALSE
)
validation <- read.table(
    "Validation_dataset/PREFER_registry_subjects_GIA_results.txt",
    header = TRUE, sep = "\t", check.names = FALSE
)
patient.df <- data.frame(read_xlsx(
    "Validation_dataset/PREFER_registry_subject_data/PREFER_registry_subjects_de_id_patient_data.x
    na = c("NULL", "NA", "N/A")
))
```

```
variant.df <- data.frame(read_xlsx(
    "Validation_dataset/PREFER_registry_subject_data/PREFER_registry_subjects_de_id_genomic_result
    na = c("NULL", "NA", "N/A")
))
seqs.df <- data.frame(read_xlsx(
    "Validation_dataset/PREFER_registry_subject_data/mapped_seq_count.xlsx",
    na = c("NULL", "NA", "N/A")
))
validation <- merge(
    merge(patient.df, validation, by = "de_id"),
    seqs.df[!duplicated(seqs.df$de_id), ],
    by = "de_id"
)

# include hispanic or latino among races
validation$Race[validation$Ethnicity == "Hispanic or Latino"] <- "Hispanic or Latino"

# add ethnicity information to Asian races
validation$Race[validation$Race == "Asian" & !is.na(validation$Race)] <- paste(
    validation$Race[validation$Race == "Asian" & !is.na(validation$Race)], " - ",
    validation$Ethnicity[validation$Race == "Asian" & !is.na(validation$Race)],
    sep = ""
)

# order factor levels of race by decreasing N
validation$Race <- factor(
    validation$Race,
    levels = names(table(validation$Race)[order(table(validation$Race), decreasing = TRUE)])
)

# make sure factor levels of age bins are in the correct order
validation$patient_age_cat <- factor(
    validation$patient_age_cat,
    levels = c(
        sort(unique(validation$patient_age_cat))[-1],
        sort(unique(validation$patient_age_cat))[1]
    )
)

# collapse sub-groups of clinical stages
validation$stage[grep("Stage IV|Metastatic", validation$stage)] <- "Stage IV"
validation$stage[grep("Stage III", validation$stage)] <- "Stage III"
validation$stage[
    grepl("Stage II", validation$stage) & validation$stage != "Stage III"
] <- "Stage II"
validation$stage[
    grepl("Stage I", validation$stage) &
        validation$stage != "Stage IV" &
        validation$stage != "Stage III" &
        validation$stage != "Stage II"
] <- "Stage I"
validation$stage[
```

```
    is.na(validation$stage) | !grepl("Stage I", validation$stage)
] <- "Unknown"

# re-create TMB interpretation with very high/high/low, log transform TMB,
# and make sure to mask TMB score for those that failed testing
validation$TMB_interp <- NA
validation$TMB_interp[validation$TMB >= 10] <- "High ( 10)"
validation$TMB_interp[validation$TMB < 10] <- "Not high (<10)"
validation$TMB_interp[
    validation$de_id %in% validation$de_id[validation$DNA_TMB %in% c("Fail", "Not Performed")]
] <- NA
validation$TMB[
    validation$de_id %in% validation$de_id[validation$DNA_TMB %in% c("Fail", "Not Performed")]
] <- NA

# re-create PD-L1 with high/low/negative and make sure to mask PD-L1 score for those
# that failed testing
validation$PD_L1_IHC_interp <- NA
validation$PD_L1_IHC_interp[
    validation$PD_L1_IHC_result >= 50
] <- "High ( 50%)"
validation$PD_L1_IHC_interp[
    validation$PD_L1_IHC_result < 50 &
        validation$PD_L1_IHC_result >= 1
] <- "Low (1-49%)"
validation$PD_L1_IHC_interp[
    validation$PD_L1_IHC_result == 0
] <- "Negative (<1%)"
validation$PD_L1_IHC_interp[
    validation$de_id %in%
        validation$de_id[validation$PD_L1_IHC_22C3 %in% c("Fail", "Not Performed")]
] <- NA
validation$PD_L1_IHC_result[
    validation$de_id %in%
        validation$de_id[validation$PD_L1_IHC_22C3 %in% c("Fail", "Not Performed")]
] <- NA
validation$PD_L1_IHC_interp <- factor(
    validation$PD_L1_IHC_interp,
    levels = c("Negative (<1%)", "Low (1-49%)", "High ( 50%)")
)

# recode MSI variable and make those without MSI or failed testing NA for MSI
validation$MSI[validation$MSI == "MSI_H"] <- "MSI High"
validation$MSI[validation$MSI == "MSS"] <- "Stable"
validation$MSI[validation$MSI == "FT"] <- NA
validation$MSI[validation$MSI == ""] <- NA

# split lung cancers into small cell and non-small cell lung cancers
validation$omnidisease_fullname[validation$omnidisease_fullname == "Lung Cancer"] <-
    ifelse(
        grepl(
            "Small cell",
```

```r
            validation$mcode_path[validation$omnidisease_fullname == "Lung Cancer"]
        ), "Small Cell Lung Cancer", "Non-Small Cell Lung Cancer"
    )

# group cancers with <5 cases as "Other cancers"
validation$omnidisease_fullname[
    validation$omnidisease_fullname %in%
        names(table(validation$omnidisease_fullname))[table(validation$omnidisease_fullname) < 5]
] <- "Other Cancer"
validation$omnidisease_fullname <- factor(
    validation$omnidisease_fullname,
    levels = c(
        names(sort(
            table(validation$omnidisease_fullname),
            decreasing = TRUE
        ))[-which(names(sort(
            table(validation$omnidisease_fullname),
            decreasing = TRUE
        )) == "Other Cancer")],
        "Other Cancer"
    )
)

# consolidate neoplastic cells per slide and cellularity information where needed
validation$neoplastic_cells_per_slide[
    validation$neoplastic_cells_per_slide == "1000-1999"
] <- ">=1000"
validation$neoplastic_cells_per_slide[
    !(validation$neoplastic_cells_per_slide %in% c(">=1000", ">=2000"))
] <- "<1000"

validation$cellularity[validation$cellularity <= 2] <- "<=2"
validation$cellularity[validation$cellularity > 2] <- ">2"

# create hard call column for ADMIXTURE results
validation$admix_GIA <- gsub("CAS_SIB", "CAS/SIB", apply(
    validation[, c("EUR", "EAS", "AMR", "SAS", "AFR", "MEA", "CAS_SIB")], 1,
    function(x) {
        ifelse(
            sum(x > 0.54) > 0,
            colnames(validation[
                , c("EUR", "EAS", "AMR", "SAS", "AFR", "MEA", "CAS_SIB")
            ])[x > 0.54],
            "Mixed Ancestry"
        )
    }
))

# create variable to denote who had CNV or fusions detected or not
validation$cnv <- ifelse(
    validation$de_id %in% variant.df$de_id[variant.df$variant_type == "CNV"],
    "Positive",
```

```r
        ifelse(
            validation$de_id %in% patient.df$de_id[patient.df$DNA_CNV %in% c("Pass", "Limited")],
            "Negative", NA
        )
)
validation$fusions <- ifelse(
    validation$de_id %in% variant.df$de_id[variant.df$variant_type == "Fusion"],
    "Positive",
    ifelse(
        validation$de_id %in% patient.df$de_id[patient.df$DNA_CNV %in% c("Pass", "Limited")],
        "Negative", NA
    )
)

# create variables for whether or not GIA calls matched given 1000G population
validation$knn_match <- validation$Superpopulation_code == validation$ML_GIA
validation$cor_match <- validation$Superpopulation_code == validation$correlation_GIA
validation$admix_match <- validation$Superpopulation_code == validation$admix_GIA
validation$gia_match <- validation$Superpopulation_code == validation$consensus_GIA

# sort consensus GIA to have Mixed Ancestry and Inconclusive last
validation$consensus_GIA <- factor(
    validation$consensus_GIA,
    levels = c(
        names(table(validation$consensus_GIA))[
            !(names(table(validation$consensus_GIA)) %in% c("Mixed Ancestry", "Inconclusive"))
        ],
        "Mixed Ancestry", "Inconclusive"
    )
)
```

2.2 Technical validation

2.2.1 Patient characteristics of validation cohort

```r
# create result data.frame
results <- data.frame()
results <- rbind(
    results,
    data.frame(
        Variable = "Variable",
        N = "N", `Summary stats` = "Summary stats",
        check.names = FALSE
    )
)

# get total number of patients with and without self-reported race data
results <- rbind(
    results,
    data.frame(
```

```
        Variable = "Total number of patients",
        N = nrow(validation), `Summary stats` = "-",
        check.names = FALSE
    )
)

### self-reported race

# get totals for all patients
var <- "Race"
data.df <- table(validation[, var], rep("0", nrow(validation)))

# add results to result data.frame
results <- rbind(
    results,
    data.frame(
        Variable = c("Self-reported race and ethnicity (N, %)", rownames(data.df)),
        N = c(sum(data.df), rep("", nrow(data.df))),
        `Summary stats` = c(
            "",
            paste(
                rowSums(data.df), " ",
                "(", round(rowSums(data.df) / sum(data.df) * 100, 1), "%", ")",
                sep = ""
            )
        ),
        check.names = FALSE
    )
)

### sex

# get totals for all patients
var <- "patient_gender"
data.df <- table(
    validation[validation$patient_gender != "Unspecified", var],
    rep("0", nrow(validation[validation$patient_gender != "Unspecified", ]))
)

# add results to result data.frame
results <- rbind(
    results,
    data.frame(
        Variable = c("Sex (N, %)", rownames(data.df)),
        N = c(sum(data.df), rep("", nrow(data.df))),
        `Summary stats` = c(
            "",
            paste(
                rowSums(data.df), " ",
                "(", round(rowSums(data.df) / sum(data.df) * 100, 1), "%", ")",
                sep = ""
            )
```

```r
        ),
        check.names = FALSE
    )
)

### age

# add results to result data.frame
var <- "patient_age"
results <- rbind(
    results,
    data.frame(
        Variable = "Age (Mean±SD)",
        N = nrow(validation[!is.na(validation[, var]), ]),
        `Summary stats` = paste(
            round(mean(na.omit(validation[, var])), 1),
            round(sd(na.omit(validation[, var])), 1),
            sep = "±"
        ),
        check.names = FALSE
    )
)

### age group

# get totals for all patients
var <- "patient_age_cat"
data.df <- table(validation[, var], rep("0", nrow(validation)))

# add results to result data.frame
results <- rbind(
    results,
    data.frame(
        Variable = c("Age group (N, %)", rownames(data.df)),
        N = c(sum(data.df), rep("", nrow(data.df))),
        `Summary stats` = c(
            "",
            paste(
                rowSums(data.df), " ",
                "(", round(rowSums(data.df) / sum(data.df) * 100, 1), "%", ")",
                sep = ""
            )
        ),
        check.names = FALSE
    )
)

### cancer type

# get totals for all patients
var <- "omnidisease_fullname"
data.df <- table(validation[, var], rep("0", nrow(validation)))
```

```
# add results to result data.frame
results <- rbind(
    results,
    data.frame(
        Variable = c("Cancer type (N, %)", rownames(data.df)),
        N = c(sum(data.df), rep("", nrow(data.df))),
        `Summary stats` = c(
            "",
            paste(
                rowSums(data.df), " ",
                "(", round(rowSums(data.df) / sum(data.df) * 100, 1), "%", ")",
                sep = ""
            )
        ),
        check.names = FALSE
    )
)

### clinical stage

# get totals for all patients
var <- "stage"
data.df <- table(
    validation[validation[, var] != "Unknown", var],
    rep("0", nrow(validation[validation[, var] != "Unknown", ]))
)

# add results to result data.frame
results <- rbind(
    results,
    data.frame(
        Variable = c("Known clinical stage (N, %)", rownames(data.df)),
        N = c(sum(data.df), rep("", nrow(data.df))),
        `Summary stats` = c(
            "",
            paste(
                rowSums(data.df), " ",
                "(", round(rowSums(data.df) / sum(data.df) * 100, 1), "%", ")",
                sep = ""
            )
        ),
        check.names = FALSE
    )
)

### tumor specimen location

# get totals for all patients
var <- "cancer_type"
data.df <- table(
    validation[validation[, var] != "Recurrent", var],
```

```r
    rep("0", nrow(validation[validation[, var] != "Recurrent", ]))
)

# add results to result data.frame
results <- rbind(
    results,
    data.frame(
        Variable = c("Tumor specimen location (N, %)", rownames(data.df)),
        N = c(sum(data.df), rep("", nrow(data.df))),
        `Summary stats` = c(
            "",
            paste(
                rowSums(data.df), " ",
                "(", round(rowSums(data.df) / sum(data.df) * 100, 1), "%", ")",
                sep = ""
            )
        ),
        check.names = FALSE
    )
)

### TMB score (mut/Mb)

# add results to result data.frame
var <- "TMB"
results <- rbind(
    results,
    data.frame(
        Variable = "TMB (Mut/Mb) (Mean±SD)",
        N = nrow(validation[!is.na(validation[, var]), ]),
        `Summary stats` = paste(
            round(mean(na.omit(validation[, var])), 1),
            round(sd(na.omit(validation[, var])), 1),
            sep = "±"
        ),
        check.names = FALSE
    )
)

### TMB high, not high

# get totals for all patients
var <- "TMB_interp"
data.df <- table(validation[, var], rep("0", nrow(validation)))

# add results to result data.frame
results <- rbind(
    results,
    data.frame(
        Variable = c("TMB level (N, %)", rownames(data.df)),
        N = c(sum(data.df), rep("", nrow(data.df))),
        `Summary stats` = c(
```

```
            "",
            paste(
                rowSums(data.df), " ",
                "(", round(rowSums(data.df) / sum(data.df) * 100, 1), "%", ")",
                sep = ""
            )
        ),
        check.names = FALSE
    )
)

### MSI level high or stable

# get totals for all patients
var <- "MSI"
data.df <- table(validation[, var], rep("0", nrow(validation)))

# add results to result data.frame
results <- rbind(
    results,
    data.frame(
        Variable = c("MSI level (N, %)", rownames(data.df)),
        N = c(sum(data.df), rep("", nrow(data.df))),
        `Summary stats` = c(
            "",
            paste(
                rowSums(data.df), " ",
                "(", round(rowSums(data.df) / sum(data.df) * 100, 1), "%", ")",
                sep = ""
            )
        ),
        check.names = FALSE
    )
)

### Neoplastic cells per slide

# get totals for all patients
var <- "neoplastic_cells_per_slide"
data.df <- table(validation[, var], rep("0", nrow(validation)))

# add results to result data.frame
results <- rbind(
    results,
    data.frame(
        Variable = c("Neoplastic cells per slide (N, %)", rownames(data.df)),
        N = c(sum(data.df), rep("", nrow(data.df))),
        `Summary stats` = c(
            "",
            paste(
                rowSums(data.df), " ",
                "(", round(rowSums(data.df) / sum(data.df) * 100, 1), "%", ")",
```

```r
                sep = ""
            )
        ),
        check.names = FALSE
    )
)

### Tumor specimen cellularity

# get totals for all patients
var <- "cellularity"
data.df <- table(validation[, var], rep("0", nrow(validation)))

# add results to result data.frame
results <- rbind(
    results,
    data.frame(
        Variable = c("Tumor specimen cellularity (N, %)", rownames(data.df)),
        N = c(sum(data.df), rep("", nrow(data.df))),
        `Summary stats` = c(
            "",
            paste(
                rowSums(data.df), " ",
                "(", round(rowSums(data.df) / sum(data.df) * 100, 1), "%", ")",
                sep = ""
            )
        ),
        check.names = FALSE
    )
)

### write out results

# create workbook
wb <- createWorkbook()

# add worksheet, write data, and format output
addWorksheet(wb, "Patient characteristics")

writeData(wb, "Patient characteristics", results, keepNA = TRUE, colNames = FALSE)

setColWidths(
    wb, "Patient characteristics",
    cols = seq_len(ncol(results)),
    widths = c(41, 5, 15)
)

addStyle(
    wb, "Patient characteristics",
    cols = seq_len(ncol(results)), rows = 1:(nrow(results) + 1),
    gridExpand = TRUE, style = left_just, stack = TRUE
)
```

```r
addStyle(
    wb, "Patient characteristics",
    cols = 1,
    rows = c(
        which(results$Variable == levels(validation$Race)[1]):
        which(results$Variable == levels(validation$Race)[length(levels(validation$Race))]),
        which(results$Variable == "Female"):which(results$Variable == "Male"),
        which(results$Variable == " 40"):which(results$Variable == ">90"),
        which(results$Variable == levels(validation$omnidisease_fullname)[1]):
        which(
            results$Variable ==
                levels(validation$omnidisease_fullname)[
                    length(levels(validation$omnidisease_fullname))
                ]
        ),
        which(results$Variable == "Stage II"):which(results$Variable == "Stage IV"),
        which(results$Variable == "Metastatic"):which(results$Variable == "Primary"),
        which(results$Variable == "High ( 10)"):which(results$Variable == "Not high (<10)"),
        which(results$Variable == "MSI High"):which(results$Variable == "Stable"),
        which(results$Variable == "<1000"):which(results$Variable == ">=2000"),
        which(results$Variable == "<=2"):which(results$Variable == ">2")
    ),
    gridExpand = TRUE, style = center, stack = TRUE
)

addStyle(
    wb, "Patient characteristics",
    cols = seq_len(ncol(results)), rows = 1,
    gridExpand = TRUE, style = bold, stack = TRUE
)

addStyle(
    wb, "Patient characteristics",
    cols = seq_len(ncol(results)),
    rows = c(1, 2, (nrow(results) + 1)), gridExpand = TRUE,
    style = horizontal_border_med, stack = TRUE
)

addStyle(
    wb, "Patient characteristics",
    cols = seq_len(ncol(results)),
    rows = c(
        grep("race", results$Variable),
        grep("Sex", results$Variable),
        grep("Age \\(", results$Variable),
        grep("Age group", results$Variable),
        grep("Cancer type", results$Variable),
        grep("Known", results$Variable),
        grep("Tumor specimen", results$Variable),
        grep("TMB", results$Variable),
        grep("MSI level", results$Variable),
```

```
        grep("Neoplastic", results$Variable),
        grep("cellularity", results$Variable)
    ),
    gridExpand = TRUE, style = horizontal_border_thin, stack = TRUE
)

# save workbook
saveWorkbook(
    wb,
    "Validation_dataset/GIA_technical_validation_results/Patient_characteristics.xlsx",
    overwrite = TRUE
)
```

2.2.2 Sequence coverage

```
# test for differences in mapped sequences
g <- ggplot(
    validation[validation$consensus_GIA != "Inconclusive", ],
    aes(x = consensus_GIA, y = log(mapped_seqs), fill = consensus_GIA)
) +
    geom_boxplot() +
    scale_fill_manual(
        values = c(labcorp.col[c(1, 3, 6, 7, 4, 8, 2)], "black")
    ) +
    stat_pwc(p.adjust.method = "none") +
    guides(fill = "none") +
    labs(x = "Consensus GIA", y = "log(Mapped sequence count)") +
    theme_bw() +
    theme(text = element_text(size = 16))
ggsave(
    "Validation_dataset/GIA_technical_validation_results/Seq_N_differences.pdf",
    g,
    device = "pdf", width = 10, height = 10
)
```

2.2.3 Technical validation results

2.2.3.1 Relationship between self-reported race/ethnicity and consensus GIA calls

```
# get race labels with Ns
validation$Race_N <- ifelse(
    validation$Race == "White",
    paste("White\nN=", sum(na.omit(validation$Race == "White")), sep = ""),
    ifelse(
        validation$Race == "Black or African American",
        paste(
            "Black or\nAfrican American\nN=",
            sum(na.omit(validation$Race == "Black or African American")),
            sep = ""
```

```
        ),
        ifelse(
            validation$Race == "Hispanic or Latino",
            paste(
                "Hispanic\nor Latino\nN=",
                sum(na.omit(validation$Race == "Hispanic or Latino")),
                sep = ""
            ),
            ifelse(
                validation$Race == "American Indian or Alaska Native",
                paste(
                    "American Indian\nor Alaska Native\nN=",
                    sum(na.omit(validation$Race == "American Indian or Alaska Native")),
                    sep = ""
                ),
                ifelse(
                    validation$Race == "Asian - Indian",
                    paste(
                        "Asian - Indian N=",
                        sum(na.omit(validation$Race == "Asian - Indian")),
                        sep = ""
                    ),
                    ifelse(
                        validation$Race == "Asian - Vietnamese",
                        paste(
                            "Asian - Vietnamese N=",
                            sum(na.omit(validation$Race == "Asian - Vietnamese")),
                            sep = ""
                        ), NA
                    )
                )
            )
        )
    )
)
validation$Race_N <- factor(
    validation$Race_N,
    levels = names(
        table(validation$Race_N)[order(table(validation$Race_N), decreasing = TRUE)]
    )
)

# create sankey (alluvial) chart showing relation between self-reported race and GIA
plot.data <- ddply(
    validation[!is.na(validation$Superpopulation_code), ],
    .(Race_N, consensus_GIA), summarize,
    freq = log(length(order_id) + 1)
)
plot.data$consensus_GIA <- ifelse(
    plot.data$consensus_GIA == "EUR",
    paste(
        "European ancestry\nN=",
```

```
        sum(
            validation[
                !is.na(validation$Superpopulation_code),
            ]$consensus_GIA == "EUR"
        ),
        sep = ""
    ),
    ifelse(
        plot.data$consensus_GIA == "AFR",
        paste(
            "African ancestry\nN=",
            sum(
                validation[
                    !is.na(validation$Superpopulation_code),
                ]$consensus_GIA == "AFR"
            ),
            sep = ""
        ),
        ifelse(
            plot.data$consensus_GIA == "AMR",
            paste(
                "American ancestry\nN=",
                sum(
                    validation[
                        !is.na(validation$Superpopulation_code),
                    ]$consensus_GIA == "AMR"
                ),
                sep = ""
            ),
            ifelse(
                plot.data$consensus_GIA == "EAS",
                paste(
                    "East Asian ancestry N=",
                    sum(
                        validation[
                            !is.na(validation$Superpopulation_code),
                        ]$consensus_GIA == "EAS"
                    ),
                    sep = ""
                ),
                ifelse(
                    plot.data$consensus_GIA == "SAS",
                    paste(
                        "South Asian ancestry N=",
                        sum(
                            validation[
                                !is.na(validation$Superpopulation_code),
                            ]$consensus_GIA == "SAS"
                        ),
                        sep = ""
                    ),
                    ifelse(
```

```
                    plot.data$consensus_GIA == "MEA",
                    paste(
                        "Middle Eastern ancestry\nN=",
                        sum(
                            validation[
                                !is.na(validation$Superpopulation_code),
                            ]$consensus_GIA == "MEA"
                        ),
                        sep = ""
                    ),
                    ifelse(
                        plot.data$consensus_GIA == "CAS/SIB",
                        paste(
                            "Central Asian/Siberian\nancestry N=",
                            sum(
                                validation[
                                    !is.na(validation$Superpopulation_code),
                                ]$consensus_GIA == "CAS/SIB"
                            ),
                            sep = ""
                        ),
                        ifelse(
                            plot.data$consensus_GIA == "Mixed Ancestry",
                            paste(
                                "Mixed ancestry\nN=",
                                sum(
                                    validation[
                                        !is.na(validation$Superpopulation_code),
                                    ]$consensus_GIA == "Mixed Ancestry"
                                ),
                                sep = ""
                            ),
                            ifelse(
                                plot.data$consensus_GIA == "Inconclusive",
                                paste(
                                    "Inconclusive\nN=",
                                    sum(
                                        validation[
                                            !is.na(validation$Superpopulation_code),
                                        ]$consensus_GIA == "Inconclusive"
                                    ),
                                    sep = ""
                                ),
                                NA
                            )
                        )
                    )
                )
            )
        )
    )
)
```

```r
)
plot.data$consensus_GIA <- factor(
    plot.data$consensus_GIA,
    levels = c(
        sort(
            unique(
                plot.data$consensus_GIA[
                    grep("Mixed|Inconclusive", plot.data$consensus_GIA, invert = TRUE)
                ]
            )
        ),
        sort(
            unique(
                plot.data$consensus_GIA[
                    grep("Mixed|Inconclusive", plot.data$consensus_GIA)
                ]
            ),
            decreasing = TRUE
        )
    )
)
labcorp.col <- c(
    "#3a5ce9", "#4cd5f7", "#f7758c", "#918f90",
    "#1a2188", "#2998e3", "#6c2fac", "#b4f6f5"
)
g <- ggplot(plot.data, aes(axis1 = Race_N, axis2 = consensus_GIA, y = freq)) +
    geom_flow(aes(fill = consensus_GIA), width = 1 / 4, alpha = 0.5) +
    geom_stratum(
        width = 1 / 4, color = "black",
        fill = c(
            rev(c(labcorp.col[c(4, 1, 6, 3, 2, 7)])),
            rev(c(labcorp.col[c(1, 3, 6, 7, 4, 8, 2)], "black", labcorp.col[5]))
        )
    ) +
    geom_label(stat = "stratum", aes(label = after_stat(stratum)), size = 4) +
    scale_x_discrete(limits = c("Race_N", "consensus_GIA"), expand = c(0, 0.5)) +
    scale_fill_manual(
        values = c(labcorp.col[c(1, 3, 6, 7, 4, 8, 2)], "black", labcorp.col[5])
    ) +
    theme_void() +
    theme(legend.position = "none")
ggsave(
    "Validation_dataset/GIA_technical_validation_results/Race_vs_GIA.pdf",
    g,
    device = "pdf", width = 7, height = 10
)
```

2.2.3.2 Projection of patient genetic PCs on reference sample PCs

```r
# import reference and patient PCs
pcs <- data.frame()
for (i in list.files("Validation_dataset/GIA_results/", pattern = "P-")) {
    id <- sapply(
        i, function(x) {
            paste(
                strsplit(x, "\\-")[[1]][1],
                strsplit(x, "\\-")[[1]][2],
                strsplit(x, "\\-")[[1]][3],
                sep = "-"
            )
        }
    )
    if (id %in% validation$order_id) {
        patient.pcs <- read.table(
            paste(
                "Validation_dataset/GIA_results/", i,
                "/PC_based_classifier/PCA_results.eigenvec",
                sep = ""
            ),
            header = TRUE, sep = "\t", comment.char = ""
        )[, -1]
        patient.pcs$IID[grep("P\\-", patient.pcs$IID)] <- id
        patient.pcs <- merge(
            patient.pcs, ref.pops[, c("SequenceID", "Superpopulation code")],
            by = 1, all.x = TRUE
        )
        patient.pcs$`Superpopulation code`[is.na(patient.pcs$`Superpopulation code`)] <-
            as.character(validation$consensus_GIA[validation$order_id == id])
        if (median(patient.pcs$PC1[patient.pcs$`Superpopulation code` == "EUR"]) < 0) {
            patient.pcs$PC1 <- patient.pcs$PC1 * -1
        }
        if (median(patient.pcs$PC2[patient.pcs$`Superpopulation code` == "EUR"]) < 0) {
            patient.pcs$PC2 <- patient.pcs$PC2 * -1
        }
        pcs <- rbind(pcs, patient.pcs)
    }
}
pcs <- plyr::ddply(
    pcs[pcs$`Superpopulation code` != "Inconclusive", ], .(IID), summarize,
    IID = unique(IID), PC1 = median(PC1), PC2 = median(PC2),
    `Superpopulation code` = unique(`Superpopulation code`)
)
pcs$Dataset <- "Reference"
pcs$Dataset[grep("P\\-", pcs$IID)] <- "Patient"

# create PC plot of reference and patient samples
g <- ggplot(
    data = pcs[pcs$Dataset != "Patient", ],
    aes(x = PC1, y = PC2, color = `Superpopulation code`, shape = Dataset)
) +
    geom_point(size = 3, alpha = 0.5) +
```

```
    geom_point(
        data = pcs[
            pcs$Dataset == "Patient" &
                pcs$`Superpopulation code` %in% c("EUR", "AFR", "AMR"),
        ],
        aes(x = PC1, y = PC2, fill = `Superpopulation code`), size = 3, color = "black"
    ) +
    geom_point(
        data = pcs[
            pcs$Dataset == "Patient" &
                pcs$`Superpopulation code` %in%
                    c("MEA", "SAS", "EAS", "CAS/SIB", "OCN", "Mixed Ancestry"),
        ],
        aes(x = PC1, y = PC2, fill = `Superpopulation code`), size = 3, color = "black"
    ) +
    scale_color_manual(values = c(labcorp.col[c(1, 3, 6, 7, 4, 8)], "gold", labcorp.col[2])) +
    scale_fill_manual(
        values = c(labcorp.col[c(1, 3, 6, 7, 4, 8)], "black", labcorp.col[2])
    ) +
    scale_shape_manual(values = c(23, 19)) +
    theme_bw() +
    theme(text = element_text(size = 18), legend.title = element_blank()) +
    guides(fill = "none")
ggsave(
    "Validation_dataset/GIA_technical_validation_results/PCA.pdf", g,
    device = "pdf", width = 10, height = 10
)
write.table(
    pcs, "Validation_dataset/GIA_technical_validation_results/PCA.txt",
    row.names = FALSE, quote = FALSE, sep = "\t"
)
```

2.2.3.3 ADMIXTURE ancestral fractions

```
# visualize ADMIXTURE fractions for each patient
plot.data <- reshape2::melt(
    validation[
        validation$consensus_GIA == "EUR",
        c("order_id", "EUR", "AFR", "AMR", "MEA", "SAS", "EAS", "CAS_SIB", "consensus_GIA")
    ]
)
plot.data <- plot.data[order(plot.data$value, decreasing = TRUE), ]
plot.data$order_id <- factor(plot.data$order_id, levels = unique(plot.data$order_id))
plot.data$value[plot.data$value > 0] <- plot.data$value[plot.data$value > 0] + 0.005
g1 <- ggplot(plot.data, aes(y = value, x = order_id, fill = variable)) +
    geom_bar(stat = "identity", width = 1) +
    coord_flip(ylim = c(0, 1), expand = FALSE) +
    facet_grid(consensus_GIA ~ ., scales = "free_y", space = "free_y", switch = "y") +
    scale_fill_manual(values = c(labcorp.col[c(4, 1, 3, 8, 2, 7, 6)])) +
    theme_bw() +
    theme(
```

```r
        text = element_text(size = 20),
        axis.text.y = element_blank(),
        axis.ticks.y = element_blank(),
        strip.text.y.left = element_text(angle = 0),
        strip.background = element_rect(fill = "grey90", color = "black"),
        legend.title = element_blank(),
        panel.grid.major = element_blank(),
        panel.grid.minor = element_blank()
    ) +
    labs(x = "", y = "")

plot.data <- reshape2::melt(
    validation[
        !(validation$consensus_GIA %in% c("EUR", "Inconclusive")),
        c("order_id", "EUR", "AFR", "AMR", "MEA", "SAS", "EAS", "CAS_SIB", "consensus_GIA")
    ]
)
plot.data <- plot.data[order(plot.data$value, decreasing = TRUE), ]
plot.data$order_id <- factor(plot.data$order_id, levels = unique(plot.data$order_id))
plot.data$consensus_GIA <- factor(
    gsub("Mixed Ancestry", "Mixed", plot.data$consensus_GIA),
    levels = c("EUR", "AFR", "AMR", "CAS/SIB", "MEA", "SAS", "EAS", "Mixed")
)
plot.data$value[plot.data$value > 0] <- plot.data$value[plot.data$value > 0] + 0.005
g2 <- ggplot(plot.data, aes(y = value, x = order_id, fill = variable)) +
    geom_bar(stat = "identity", width = 1) +
    coord_flip(ylim = c(0, 1), expand = FALSE) +
    facet_grid(consensus_GIA ~ ., scales = "free_y", space = "free_y", switch = "y") +
    scale_fill_manual(values = c(labcorp.col[c(4, 1, 3, 8, 2, 7, 6)])) +
    theme_bw() +
    theme(
        text = element_text(size = 20),
        axis.text.y = element_blank(),
        axis.ticks.y = element_blank(),
        strip.text.y.left = element_text(angle = 0),
        strip.background = element_rect(fill = "grey90", color = "black"),
        legend.title = element_blank(),
        panel.grid.major = element_blank(),
        panel.grid.minor = element_blank()
    ) +
    labs(x = "", y = "")
g <- ggarrange(
    g1, NULL, g2, NULL,
    ncol = 4, nrow = 1, widths = c(1, 0.007, 1, 0.007), common.legend = TRUE
)
g <- annotate_figure(
    g,
    bottom = textGrob("ADMIXTURE fraction", gp = gpar(cex = 2), vjust = -1)
)
ggsave(
    "Validation_dataset/GIA_technical_validation_results/ADMIXTURE_fractions.pdf", g,
    device = "pdf", width = 10, height = 25
```

```r
)

# plot distribution of ADMIXTURE fractions within GIA groups
plot.data <- reshape2::melt(
    validation[
        !(validation$consensus_GIA %in% c("Mixed Ancestry", "Inconclusive")),
        c("order_id", "AFR", "AMR", "CAS_SIB", "EAS", "EUR", "MEA", "SAS", "consensus_GIA")
    ]
)
plot.data$variable <- gsub("CAS_SIB", "CAS/SIB", plot.data$variable)
g <- ggplot(plot.data, aes(x = variable, y = value, color = variable, fill = variable)) +
    geom_boxplot(color = "black", alpha = 0.25, outlier.size = 0) +
    geom_point(size = 3, alpha = 0.75) +
    facet_grid(~consensus_GIA) +
    scale_color_manual(values = labcorp.col[c(1, 3, 6, 7, 4, 8, 2)]) +
    scale_fill_manual(values = labcorp.col[c(1, 3, 6, 7, 4, 8, 2)]) +
    guides(color = "none", fill = "none") +
    labs(x = "Ancestry populations", y = "ADMIXTURE fraction") +
    theme_bw() +
    theme(
        text = element_text(size = 14),
        axis.text.x = element_text(size = 10, angle = 30, hjust = 1)
    )
ggsave(
    "Validation_dataset/GIA_technical_validation_results/ADMIXTURE_fraction_distributions.pdf",
    g,
    device = "pdf", width = 16, height = 4
)

# plot distribution of ADMIXTURE fractions of Native American ethnicity
plot.data <- reshape2::melt(
    validation[
        !(validation$consensus_GIA %in% c("Mixed Ancestry", "Inconclusive")) &
            (validation$Ethnicity == "Native American" | is.na(validation$Ethnicity)) &
            validation$Race == "White",
        c("order_id", "AFR", "AMR", "CAS_SIB", "EAS", "EUR", "MEA", "SAS", "Ethnicity")
    ]
)
plot.data$Ethnicity[!is.na(plot.data$Ethnicity)] <- "Native American ethnicity"
plot.data$Ethnicity[is.na(plot.data$Ethnicity)] <- "No ethnicity"
plot.data$variable <- gsub("CAS_SIB", "CAS/SIB", plot.data$variable)
plot.p <- sapply(
    unique(plot.data$variable),
    function(x) {
        paste(
            x, " (P = ",
            round(wilcox.test(
                plot.data$value[
                    plot.data$variable == x & plot.data$Ethnicity == "Native American ethnicity"
                ],
                plot.data$value[
                    plot.data$variable == x & plot.data$Ethnicity == "No ethnicity"
```

```
                ]
            )$p.value, 2), ")",
            sep = ""
        )
    }
)
g <- ggplot(plot.data, aes(x = Ethnicity, y = value, color = Ethnicity, fill = Ethnicity)) +
    stat_summary(fun = "mean", geom = "bar") +
    stat_summary(fun.data = "mean_se", geom = "errorbar", linewidth = 1, width = 0.5) +
    facet_wrap(~variable, scales = "free", nrow = 1, labeller = labeller(variable = plot.p)) +
    scale_y_continuous(labels = scales::percent) +
    scale_x_discrete(labels = stringr::str_wrap(names(table(plot.data$Ethnicity)), 10)) +
    scale_color_manual(values = c("grey50", "black")) +
    scale_fill_manual(values = c("grey50", "black")) +
    guides(color = "none", fill = "none") +
    labs(y = "ADMIXTURE fraction (%)") +
    theme_bw() +
    theme(
        text = element_text(size = 14),
        axis.title.x = element_blank()
    )
ggsave(
    "Validation_dataset/GIA_technical_validation_results/ADMIXTURE_fraction_NatAmer_vs_not.pdf",
    g,
    device = "pdf", width = 15, height = 4
)
```

2.2.3.4 Performance metrics of GIA classification compared to self-reported race/ethnicity

```
# calculate classification performance metrics
knn <- confusionMatrix(
    data = factor(validation$ML_GIA, levels = unique(validation$ML_GIA)),
    reference = factor(
        validation$Superpopulation_code,
        levels = unique(validation$ML_GIA)
    ),
    mode = "prec_recall"
)
corr <- confusionMatrix(
    data = factor(validation$correlation_GIA, levels = unique(validation$correlation_GIA)),
    reference = factor(
        validation$Superpopulation_code,
        levels = unique(validation$correlation_GIA)
    ),
    mode = "prec_recall"
)
admix <- confusionMatrix(
    data = factor(validation$admix_GIA, levels = unique(validation$admix_GIA)),
    reference = factor(
        validation$Superpopulation_code,
        levels = unique(validation$admix_GIA)
```

```
    ),
    mode = "prec_recall"
)
consensus <- confusionMatrix(
    data = factor(validation$consensus_GIA, levels = unique(validation$consensus_GIA)),
    reference = factor(
        validation$Superpopulation_code,
        levels = unique(validation$consensus_GIA)
    ),
    mode = "prec_recall"
)

# plot performance metrics results
metrics <- c("Sensitivity", "Specificity", "Balanced Accuracy", "Precision", "F1")
plot.data <- rbind(
    data.frame(
        Method = "kNN",
        `Ancestry group` = as.vector(sapply(
            c(
                "Mean ± SD",
                colnames(knn$table)[!grepl("MEA", colnames(knn$table))]
            ),
            function(x) {
                rep(x, length(metrics))
            }
        )),
        Metric = rep(
            metrics,
            length(colnames(knn$table)[
                !grepl("MEA", colnames(knn$table))
            ]) + 1
        ),
        `Metric value` = c(
            colMeans(knn$byClass[
                !grepl("MEA", rownames(knn$byClass)), metrics
            ]),
            t(knn$byClass[
                !grepl("MEA", rownames(knn$byClass)), metrics
            ])
        ),
        lower = c(
            colMeans(knn$byClass[
                !grepl("MEA", rownames(knn$byClass)), metrics
            ]) -
                apply(knn$byClass[
                    !grepl("MEA", rownames(knn$byClass)), metrics
                ], 2, sd),
            rep(NA, length(colnames(knn$table)[
                !grepl("MEA", colnames(knn$table))
            ]) * length(metrics))
        ),
        upper = c(
```

```
        colMeans(knn$byClass[
            !grepl("MEA", rownames(knn$byClass)), metrics
        ]) +
            apply(knn$byClass[
                !grepl("MEA", rownames(knn$byClass)), metrics
            ], 2, sd),
        rep(NA, length(colnames(knn$table)[
            !grepl("MEA", colnames(knn$table))
        ]) * length(metrics))
    ),
    check.names = FALSE
),
data.frame(
    Method = "Correlation",
    `Ancestry group` = as.vector(sapply(
        c(
            "Mean ± SD",
            colnames(corr$table)[!grepl("MEA", colnames(corr$table))]
        ),
        function(x) {
            rep(x, length(metrics))
        }
    )),
    Metric = rep(
        metrics,
        length(colnames(corr$table)[
            !grepl("MEA", colnames(corr$table))
        ]) + 1
    ),
    `Metric value` = c(
        colMeans(corr$byClass[
            !grepl("MEA", rownames(corr$byClass)), metrics
        ]),
        t(corr$byClass[
            !grepl("MEA", rownames(corr$byClass)), metrics
        ])
    ),
    lower = c(
        colMeans(corr$byClass[
            !grepl("MEA", rownames(corr$byClass)), metrics
        ]) -
            apply(corr$byClass[
                !grepl("MEA", rownames(corr$byClass)), metrics
            ], 2, sd),
        rep(NA, length(colnames(corr$table)[
            !grepl("MEA", colnames(corr$table))
        ]) * length(metrics))
    ),
    upper = c(
        colMeans(corr$byClass[
            !grepl("MEA", rownames(corr$byClass)), metrics
        ]) +
```

```
            apply(corr$byClass[
                !grepl("MEA", rownames(corr$byClass)), metrics
            ], 2, sd),
        rep(NA, length(colnames(corr$table)[
            !grepl("MEA", colnames(corr$table))
        ]) * length(metrics))
    ),
    check.names = FALSE
),
data.frame(
    Method = "ADMIXTURE",
    `Ancestry group` = as.vector(sapply(
        c(
            "Mean ± SD",
            colnames(admix$table)[!grepl("MEA|Mix", colnames(admix$table))]
        ),
        function(x) {
            rep(x, length(metrics))
        }
    )),
    Metric = rep(
        metrics,
        length(colnames(admix$table)[
            !grepl("MEA|Mix", colnames(admix$table))
        ]) + 1
    ),
    `Metric value` = c(
        colMeans(admix$byClass[
            !grepl("MEA|Mix", rownames(admix$byClass)), metrics
        ]),
        t(admix$byClass[
            !grepl("MEA|Mix", rownames(admix$byClass)), metrics
        ])
    ),
    lower = c(
        colMeans(admix$byClass[
            !grepl("MEA|Mix", rownames(admix$byClass)), metrics
        ]) -
            apply(admix$byClass[
                !grepl("MEA|Mix", rownames(admix$byClass)), metrics
            ], 2, sd),
        rep(
            NA,
            length(colnames(admix$table)[
                !grepl("MEA|Mix", colnames(admix$table))
            ]) * length(metrics)
        )
    ),
    upper = c(
        colMeans(admix$byClass[
            !grepl("MEA|Mix", rownames(admix$byClass)), metrics
        ]) +
```

```
            apply(admix$byClass[
                !grepl("MEA|Mix", rownames(admix$byClass)), metrics
            ], 2, sd),
        rep(
            NA,
            length(colnames(admix$table)[
                !grepl("MEA|Mix", colnames(admix$table))
            ]) * length(metrics)
        )
    ),
    check.names = FALSE
),
data.frame(
    Method = "Consensus",
    `Ancestry group` = as.vector(sapply(
        c(
            "Mean ± SD",
            colnames(consensus$table)[
                !grepl("MEA|Mix|Incon", colnames(consensus$table))
            ]
        ),
        function(x) {
            rep(x, length(metrics))
        }
    )),
    Metric = rep(
        metrics,
        length(colnames(consensus$table)[
            !grepl("MEA|Mix|Incon", colnames(consensus$table))
        ]) + 1
    ),
    `Metric value` = c(
        colMeans(consensus$byClass[
            !grepl("MEA|Mix|Incon", rownames(consensus$byClass)), metrics
        ]),
        t(consensus$byClass[
            !grepl("MEA|Mix|Incon", rownames(consensus$byClass)), metrics
        ])
    ),
    lower = c(
        colMeans(consensus$byClass[
            !grepl("MEA|Mix|Incon", rownames(consensus$byClass)), metrics
        ]) -
            apply(consensus$byClass[
                !grepl("MEA|Mix|Incon", rownames(consensus$byClass)), metrics
            ], 2, sd),
        rep(
            NA,
            length(colnames(consensus$table)[
                !grepl("MEA|Mix|Incon", colnames(consensus$table))
            ]) * length(metrics)
        )
```

```r
        ),
        upper = c(
            colMeans(consensus$byClass[
                !grepl("MEA|Mix|Incon", rownames(consensus$byClass)), metrics
            ]) +
                apply(consensus$byClass[
                    !grepl("MEA|Mix|Incon", rownames(consensus$byClass)), metrics
                ], 2, sd),
            rep(
                NA,
                length(colnames(consensus$table)[
                    !grepl("MEA|Mix|Incon", colnames(consensus$table))
                ]) * length(metrics)
            )
        ),
        check.names = FALSE
    )
)
plot.data$Method <- factor(
    plot.data$Method,
    levels = c("kNN", "Correlation", "ADMIXTURE", "Consensus")
)
plot.data$`Ancestry group` <- factor(
    gsub("CAS_SIB", "CAS/SIB", plot.data$`Ancestry group`),
    levels = c("AFR", "AMR", "CAS/SIB", "EAS", "EUR", "SAS", "Mean ± SD")
)
plot.data$Metric <- dplyr::recode(
    factor(plot.data$Metric, levels = metrics),
    Sensitivity = "Sensitivity/Recall"
)
plot.data$upper[plot.data$upper > 1] <- 1

g <- ggplot(plot.data, aes(y = `Metric value`, x = Method, fill = Method)) +
    geom_bar(stat = "identity", position = "dodge", alpha = 0.75) +
    geom_errorbar(aes(ymin = lower, ymax = upper),
        width = 0.25, alpha = 0.75,
        position = position_dodge(0.9)
    ) +
    geom_text(
        aes(label = round(`Metric value`, 2)),
        y = min(plot.data$`Metric value`) + 0.05, size = 5
    ) +
    facet_grid(Metric ~ `Ancestry group`) +
    coord_cartesian(
        ylim = c(min(plot.data$`Metric value`), max(plot.data$`Metric value`))
    ) +
    theme_bw() +
    theme(
        text = element_text(size = 18),
        axis.text.x = element_blank(),
        axis.ticks.x = element_blank(),
        strip.background = element_rect(fill = "grey90", color = "black")
```

```
    ) +
    labs(x = "")
ggsave(
    "Validation_dataset/GIA_technical_validation_results/Performance_metric_results.pdf",
    g,
    device = "pdf", width = 16, height = 12
)
```

2.2.3.5 Concordance of GIA classification compared to self-reported race/ethnicity by cancer type and tumor characteristics

```
# calculate and plot concordances and GIA proportions for cancer types
plot.data <- ddply(
    validation, .(omnidisease_fullname), summarize,
    Concordance = round(sum(na.omit(gia_match)) / length(na.omit(gia_match)), 2)
)

g1 <- ggplot(plot.data, aes(x = omnidisease_fullname, y = Concordance)) +
    geom_col(fill = "grey") +
    geom_text(aes(label = paste(Concordance * 100, "%", sep = "")), size = 3, vjust = 2) +
    scale_y_continuous(labels = scales::percent) +
    theme_bw() +
    theme(
        axis.text.x = element_blank(),
        axis.ticks.x = element_blank(),
        axis.title.x = element_blank()
    )

g2 <- ggplot(
    validation,
    aes(
        x = omnidisease_fullname,
        fill = factor(
            consensus_GIA,
            levels = c(
                "AFR", "AMR", "CAS/SIB", "EAS", "EUR", "MEA", "SAS",
                "Mixed Ancestry", "Inconclusive"
            )
        )
    )
) +
    geom_bar(position = "fill") +
    scale_fill_manual(values = c(labcorp.col[c(1, 3, 6, 7, 4, 8, 2)], "black", labcorp.col[5])) +
    scale_y_continuous(labels = scales::percent) +
    theme_bw() +
    labs(y = "Proportion of patients") +
    theme(
        axis.text.x = element_blank(),
        axis.title.x = element_blank(),
        axis.ticks.x = element_blank(),
        legend.title = element_blank()
```

```
    )

plot.data <- ddply(
    validation[!(validation$consensus_GIA %in% c("MEA", "Mixed Ancestry", "Inconclusive")), ],
    .(omnidisease_fullname), summarize,
    Concordance = round(sum(na.omit(gia_match)) / length(na.omit(gia_match)), 2)
)

g3 <- ggplot(plot.data, aes(x = omnidisease_fullname, y = Concordance)) +
    geom_col(fill = "grey") +
    geom_text(aes(label = paste(Concordance * 100, "%", sep = "")), size = 3, vjust = 2) +
    scale_y_continuous(labels = scales::percent) +
    theme_bw() +
    labs(y = "Concordance\n(excluding non-SIRE groups)") +
    theme(
        axis.text.x = element_text(angle = 60, hjust = 1),
        axis.ticks.x = element_blank(),
        axis.title.x = element_blank()
    )

g <- ggarrange(
    g1, NULL, g2, NULL, g3,
    nrow = 5, ncol = 1, heights = c(0.2, 0, 0.4, 0, 0.4), align = "v",
    common.legend = TRUE, legend = "right"
)
ggsave(
    "Validation_dataset/GIA_technical_validation_results/Cancer_type_differences.pdf",
    g,
    device = "pdf", width = 9, height = 7
)

# test and plot differences in GIA call concordances for tumor characteristics
fish.test <- function(a, b) {
    return(fisher.test(cbind(a, b)))
}

plot.data <- ddply(
    validation[
        validation$cancer_type != "Recurrent" &
            !is.na(validation$cancer_type) & !is.na(validation$gia_match),
    ],
    .(cancer_type, gia_match), summarize,
    count = length(de_id)
)
g1 <- ggplot(plot.data, aes(x = cancer_type, y = count, fill = gia_match)) +
    geom_bar(position = "stack", stat = "identity") +
    geom_signif(
        y_position = max(plot.data$count) + 20,
        comparisons = list(c("Primary", "Metastatic")), test = "fish.test"
    ) +
    scale_fill_manual(name = "Did GIA and\nSIRE match?", values = c("black", "grey50")) +
    coord_cartesian(y = c(0, 500)) +
```

```r
    theme_bw() +
    labs(y = "Number of patient tumors", x = "Tissue specimen\nlocation") +
    theme(text = element_text(size = 14))

plot.data <- ddply(
    validation[!is.na(validation$TMB_interp) & !is.na(validation$gia_match), ],
    .(TMB_interp, gia_match), summarize,
    count = length(de_id)
)
g2 <- ggplot(plot.data, aes(x = TMB_interp, y = count, fill = gia_match)) +
    geom_bar(position = "stack", stat = "identity") +
    geom_signif(
        y_position = max(plot.data$count) + 20,
        comparisons = list(c("High ( 10)", "Not high (<10)")), test = "fish.test"
    ) +
    scale_x_discrete(labels = c(expression("" >= "10"), "< 10")) +
    scale_fill_manual(name = "Did GIA and\nSIRE match?", values = c("black", "grey50")) +
    coord_cartesian(y = c(0, 500)) +
    theme_bw() +
    labs(y = "Number of patient tumors", x = "TMB level\n(mutations/Mb)") +
    theme(
        text = element_text(size = 14),
        axis.title.y = element_blank(),
        axis.text.y = element_blank(),
        axis.ticks.y = element_blank()
    )

plot.data <- ddply(
    validation[!is.na(validation$MSI) & !is.na(validation$gia_match), ],
    .(MSI, gia_match), summarize,
    count = length(de_id)
)
plot.data <- rbind(data.frame(MSI = "MSI High", gia_match = FALSE, count = 0), plot.data)
g3 <- ggplot(plot.data, aes(x = MSI, y = count, fill = gia_match)) +
    geom_bar(position = "stack", stat = "identity") +
    geom_signif(
        y_position = max(plot.data$count) + 20,
        comparisons = list(c("MSI High", "Stable")), test = "fish.test"
    ) +
    scale_fill_manual(name = "Did GIA and\nSIRE match?", values = c("black", "grey50")) +
    coord_cartesian(y = c(0, 500)) +
    theme_bw() +
    labs(x = "MSI level") +
    theme(
        text = element_text(size = 14),
        axis.title.y = element_blank(),
        axis.text.y = element_blank(),
        axis.ticks.y = element_blank()
    )

plot.data <- ddply(
    validation[!is.na(validation$neoplastic_cells_per_slide) & !is.na(validation$gia_match), ],
```

```
    .(neoplastic_cells_per_slide, gia_match), summarize,
    count = length(de_id)
)
g4 <- ggplot(plot.data, aes(x = neoplastic_cells_per_slide, y = count, fill = gia_match)) +
    geom_bar(position = "stack", stat = "identity") +
    geom_signif(
        y_position = max(plot.data$count) + 20, step_increase = 0.1,
        comparisons = list(
            c("<1000", ">=1000"),
            c("<1000", ">=2000"),
            c(">=1000", ">=2000")
        ),
        test = "fish.test"
    ) +
    scale_x_discrete(labels = c("< 1000", expression("" >= "1000"), expression("" >= "2000"))) +
    scale_fill_manual(name = "Did GIA and\nSIRE match?", values = c("black", "grey50")) +
    coord_cartesian(y = c(0, max(plot.data$count) * 1.4)) +
    theme_bw() +
    labs(x = "Neoplastic cells\nper slide") +
    theme(
        text = element_text(size = 14),
        axis.title.y = element_blank(),
        axis.text.y = element_blank(),
        axis.ticks.y = element_blank()
    )

plot.data <- ddply(
    validation[!is.na(validation$cellularity) & !is.na(validation$gia_match), ],
    .(cellularity, gia_match), summarize,
    count = length(de_id)
)
g5 <- ggplot(plot.data, aes(x = cellularity, y = count, fill = gia_match)) +
    geom_bar(position = "stack", stat = "identity") +
    geom_signif(
        y_position = max(plot.data$count) + 20,
        comparisons = list(c("<=2", ">2")), test = "fish.test"
    ) +
    scale_x_discrete(labels = c(expression("" <= "2"), "> 2")) +
    scale_fill_manual(name = "Did GIA and\nSIRE match?", values = c("black", "grey50")) +
    coord_cartesian(y = c(0, 500)) +
    theme_bw() +
    labs(x = "Tumor specimen\ncellularity") +
    theme(
        text = element_text(size = 14),
        axis.title.y = element_blank(),
        axis.text.y = element_blank(),
        axis.ticks.y = element_blank()
    )

plot.data <- ddply(
    validation[!is.na(validation$cnv) & !is.na(validation$gia_match), ],
    .(cnv, gia_match), summarize,
```

```r
    count = length(de_id)
)
g6 <- ggplot(plot.data, aes(x = cnv, y = count, fill = gia_match)) +
    geom_bar(position = "stack", stat = "identity") +
    geom_signif(
        y_position = max(plot.data$count) + 20,
        comparisons = list(c("Negative", "Positive")), test = "fish.test"
    ) +
    scale_fill_manual(name = "Did GIA and\nSIRE match?", values = c("black", "grey50")) +
    coord_cartesian(y = c(0, 500)) +
    theme_bw() +
    labs(x = "Copy number\nalterations") +
    theme(
        text = element_text(size = 14),
        axis.title.y = element_blank(),
        axis.text.y = element_blank(),
        axis.ticks.y = element_blank()
    )

plot.data <- ddply(
    validation[!is.na(validation$fusions) & !is.na(validation$gia_match), ],
    .(fusions, gia_match), summarize,
    count = length(de_id)
)
g7 <- ggplot(plot.data, aes(x = fusions, y = count, fill = gia_match)) +
    geom_bar(position = "stack", stat = "identity") +
    geom_signif(
        y_position = max(plot.data$count) + 20,
        comparisons = list(c("Negative", "Positive")), test = "fish.test"
    ) +
    scale_fill_manual(name = "Did GIA and\nSIRE match?", values = c("black", "grey50")) +
    coord_cartesian(y = c(0, 500)) +
    theme_bw() +
    labs(x = "Gene fusions or\nrearrangements") +
    theme(
        text = element_text(size = 14),
        axis.title.y = element_blank(),
        axis.text.y = element_blank(),
        axis.ticks.y = element_blank()
    )

g <- ggarrange(
    g1, NULL, g2, NULL, g3, NULL, g4, NULL, g5, NULL, g6, NULL, g7,
    nrow = 1, ncol = 13,
    widths = c(1.2, 0, 1, 0, 1, 0, 1, 0, 1, 0, 1, 0, 1),
    align = "h", common.legend = TRUE, legend = "right"
)
ggsave(
    "Validation_dataset/GIA_technical_validation_results/Tumor_characteristic_differences.pdf",
    g,
    device = "pdf", width = 14, height = 4
)
```

3 R session information

```
R version 4.4.1 (2024-06-14 ucrt)
Platform: x86_64-w64-mingw32/x64
Running under: Windows 10 x64 (build 19045)

Matrix products: default


locale:
[1] LC_COLLATE=English_United States.utf8
[2] LC_CTYPE=English_United States.utf8
[3] LC_MONETARY=English_United States.utf8
[4] LC_NUMERIC=C
[5] LC_TIME=English_United States.utf8

time zone: America/New_York
tzcode source: internal

attached base packages:
[1] grid      stats     graphics  grDevices utils     datasets  methods
[8] base

other attached packages:
 [1] caret_6.0-94      lattice_0.22-6    ggalluvial_0.12.5 ggpubr_0.6.0
 [5] ggplot2_3.5.1     kableExtra_1.4.0  foreach_1.5.2     openxlsx_4.2.5.2
 [9] tibble_3.2.1      readxl_1.4.3      plyr_1.8.9

loaded via a namespace (and not attached):
 [1] tidyselect_1.2.1     viridisLite_0.4.2    timeDate_4032.109
 [4] farver_2.1.2         dplyr_1.1.4          fastmap_1.2.0
 [7] pROC_1.18.5          digest_0.6.36        rpart_4.1.23
[10] timechange_0.3.0     lifecycle_1.0.4      survival_3.7-0
[13] magrittr_2.0.3       compiler_4.4.1       rlang_1.1.4
[16] tools_4.4.1          utf8_1.2.4           yaml_2.3.8
[19] data.table_1.15.4    knitr_1.47           ggsignif_0.6.4
[22] labeling_0.4.3       xml2_1.3.6           abind_1.4-5
[25] withr_3.0.0          purrr_1.0.2          stats4_4.4.1
[28] nnet_7.3-19          fansi_1.0.6          e1071_1.7-14
[31] colorspace_2.1-0     future_1.33.2        globals_0.16.3
[34] scales_1.3.0         iterators_1.0.14     MASS_7.3-61
[37] cli_3.6.3            rmarkdown_2.27       ragg_1.3.2
[40] generics_0.1.3       rstudioapi_0.16.0    future.apply_1.11.2
[43] reshape2_1.4.4       proxy_0.4-27         stringr_1.5.1
[46] splines_4.4.1        parallel_4.4.1       cellranger_1.1.0
[49] vctrs_0.6.5          hardhat_1.4.0        Matrix_1.7-0
[52] jsonlite_1.8.8       carData_3.0-5        car_3.1-2
[55] rstatix_0.7.2        listenv_0.9.1        systemfonts_1.1.0
[58] gower_1.0.1          tidyr_1.3.1          recipes_1.0.10
[61] glue_1.7.0           parallelly_1.37.1    codetools_0.2-20
[64] cowplot_1.1.3        lubridate_1.9.3      stringi_1.8.4
[67] gtable_0.3.5         munsell_0.5.1        pillar_1.9.0
[70] htmltools_0.5.8.1    ipred_0.9-14         lava_1.8.0
```

```
[73] R6_2.5.1            textshaping_0.4.0   evaluate_0.24.0
[76] backports_1.5.0     broom_1.0.6         class_7.3-22
[79] Rcpp_1.0.12         zip_2.3.1           gridExtra_2.3
[82] svglite_2.1.3       nlme_3.1-165        prodlim_2024.06.25
[85] xfun_0.45           ModelMetrics_1.2.2.2 pkgconfig_2.0.3
```
